# Investigation of Proteomic and Phosphoproteomic Responses to Signaling Network Perturbations Reveals Functional Pathway Organizations in Yeast

**DOI:** 10.1016/j.celrep.2019.10.034

**Published:** 2019-11-12

**Authors:** Jiaming Li, Joao A. Paulo, David P. Nusinow, Edward L. Huttlin, Steven P. Gygi

**Affiliations:** 1 Department of Cell Biology, Harvard Medical School, Boston, MA 02115, USA; 2 Lead Contact

## Abstract

Governance of protein phosphorylation by kinases and phosphatases constitutes an essential regulatory network in eukaryotic cells. Network dysregulation leads to severe consequences and is often a key factor in disease pathogenesis. Previous studies revealed multiple roles for protein phosphorylation and pathway structures in cellular functions from different perspectives. We seek to understand the roles of kinases and phosphatases from a protein homeostasis point of view. Using a streamlined tandem mass tag (SL-TMT) strategy, we systematically measure proteomic and phosphoproteomic responses to perturbations of phosphorylation signaling networks in yeast deletion strains. Our results emphasize the requirement for protein normalization for more complete interpretation of phosphorylation data. Functional relationships between kinases and phosphatases were characterized at both proteome and phosphoproteome levels in three ways: (1) Gene Ontology enrichment analysis, (2) *Δgene-Δgene* correlation networks, and (3) molecule covariance networks. This resource illuminates kinase and phosphatase functions and pathway organizations.

## In Brief

Li et al. measure (phospho)proteomic responses to perturbations of phosphorylation signaling networks in 110 yeast deletion strains. The results emphasize that broad interpretation of phosphorylation data requires protein normalization. Kinase and phosphatase relationships are characterized at both proteome and phosphoproteome levels. Phosphorylation signaling pathway organizations are illuminated by network analyses.

## Graphical Abstract

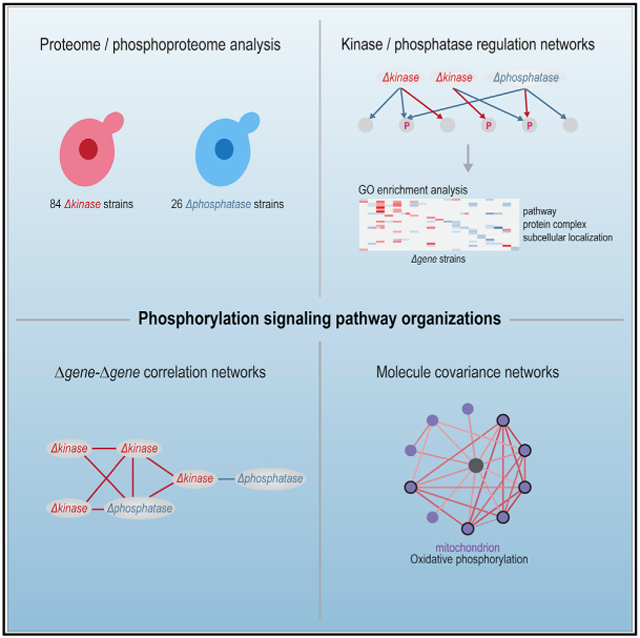

## INTRODUCTION

Protein phosphorylation signaling networks are essential regulatory guardians of cellular functions and are involved in the pathogenesis of many diseases, including cancer. Kinases and phosphatases are key components of signaling networks. Multiple roles for phosphorylation in cellular processes and pathway architectures have been uncovered. In focused studies, pathways have often been studied in isolation with different readouts, limiting the ability to gain a global view of concerted pathways. With emerging high-throughput technologies, such as RNA sequencing (RNA-seq) and mass spectrometry-based proteomics, comprehensive analyses of signaling networks can be achieved (Bodenmiller et al., 2010; Riley and Coon, 2016).

Previous efforts have used mRNA (van Wageningen et al., 2010), lipids (da Silveira Dos Santos et al., 2014), metabolites (Schulz et al., 2014), and protein phosphorylation (Bodenmiller et al., 2010) as endpoint measurements to systematically uncover the workings of many pathways in yeast using deletion strains. Deletion of a kinase, for example, silences both its kinase activity and its protein expression. Either or both of these effects might cause yeast to compensate via altered protein expression or protein activities. In phosphoproteomics, differences in measured phosphopeptide levels can be affected either in part or in whole by changes in protein abundance (Wu et al., 2011a). A full interpretation of phosphoproteomic perturbations generally requires protein abundance changes to be considered. An important previous study on phosphoproteomic analysis of yeast kinase and phosphatase mutants did not include a systematic protein abundance assay, which may limit the broad elucidation of the phosphoproteomic phenotypes (Bodenmiller et al., 2010).

Globally addressing all 518 protein kinases and 147 protein phosphatases with replicates in human cells is challenging (Bodenmiller et al., 2010). With many pathways conserved throughout evolution, yeast represents a useful model organism to study phosphorylation networks. Yeast harbors 159 genes encoding protein kinases and phosphatases and 136 of these have human homologs. In this study, we carried out a systems-level proteomic and phosphoproteomic analysis for 110 yeast single-kinase or phosphatase deletion strains under standard growth conditions. The high overlap between protein and phosphoprotein quantifications enabled the normalization of phosphorylation to account for protein abundance differences. Functional relationships between kinases and phosphatases were characterized at both proteome and phosphoproteome levels in several ways including traditional enrichment analysis, *Δgene-Δgene* correlation networks, and molecular covariance networks. Known pathways, such as high-osmolarity glycerol (HOG) and cell wall integrity (CWI) pathways, were accurately recapitulated, and potentially novel pathway architectures were suggested. The results represent a valuable resource for further investigations of kinase and phosphatase functions and regulatory organizations of signaling networks.

## RESULTS

### Proteomic and Phosphoproteomic Profiling of 110 Yeast Kinase and Phosphatase Deletion Strains

**Figure 1.**
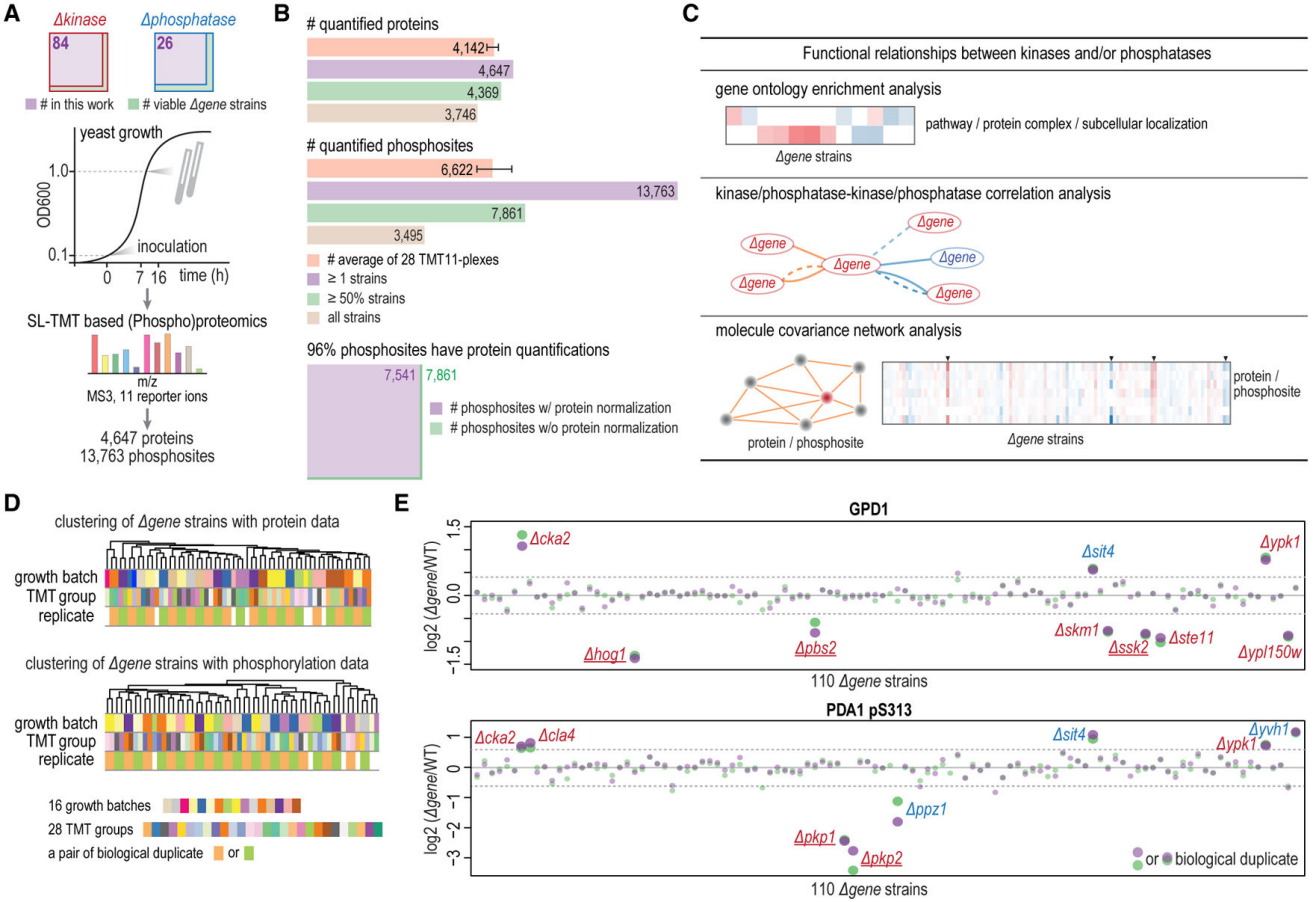
Quantitative Proteomic and Phosphoproteomic Analysis of 110 Yeast Kinase and Phosphatase Deletion Strains (A) Experimental workflow. In total, 84 kinase and 26 phosphatase deletion strains were investigated, covering 82% of all viable yeast kinase and phosphatase deletion strains. Yeast were grown in duplicate under standard conditions and harvested at optical density 600 (OD_600_) ≈ 1.0. With SL-TMT-based (phospho) proteomics, >4,600 proteins and >13,000 phosphosites were quantified. (B) Summary of the datasets. Bars show average numbers of molecules quantified per TMT11-plex and overlap of molecules quantified across 110 deletion strains. About 96% quantified phosphosites have protein quantifications and could be normalized by cognate protein ratios. Error bars indicate minimum and maximum numbers of molecules among 28 TMT11-plexes. (C) Overview of the data analyses. Functional relationships between kinases and/or phosphatases were analyzed in three ways: (1) Gene Ontology enrichment analysis, (2) *Δgene-Δgene* correlation networks, and (3) molecule covariance networks. (D) Hierarchical clustering analysis of a subset of 58 samples (see Figure S1F for full dendrogram). Biological duplicates clustered tightly. Deletion strains did not cluster with TMT groups or growth batches. (E) GPD1 (a HOG1-dependent osmostress-induced protein) showed low levels in *Δhog1, Δpbs2*, and *Δssk2*. HOG1, SSK2, and PBS2 are MAPK components of the HOG pathway. PDA1 S313 showed decreased phosphorylation levels in strains lacking its known kinases (PKP1 and PKP2). Colored dots represent measurements in biological duplicate. Dashed lines indicate 3 SD cutoff (see the STAR Methods). See also Figure S1 and Table S1.

We profiled 110 yeast strains with single deletions of nonessential genes encoding 84 kinase and 26 phosphatase catalytic subunits in duplicate, covering about 82% of all viable yeast kinase and phosphatase deletion strains (Figure 1A; Table S1). Yeast were grown in yeast-peptone-dextrose (YPD) media under standard conditions and harvested at OD_600nm_ ≈ 1.0 (Figure 1A). Gene deletions were confirmed by either proteomics (significant decreases in encoded proteins) or PCR assays (Figure S1A). Using the streamlined tandem mass tag (SL-TMT) protocol (Navarrete-Perea et al., 2018), we quantified > 4,600 proteins and > 13,000 phosphosites, both at a 1% protein-level false discovery rate (FDR) (Figure 1A).

For protein expression work, we averaged >4,100 proteins quantified per TMT11-plex with >4,300 across half of the samples (Figures 1B and S1C; Table S2). For phosphorylation work, we quantified an average of >6,600 phosphosites per TMT11-plex. Altogether, >13,000 phosphosites were captured (Figures 1B and S1D; Table S2). This dataset had a high overlap between protein data and phosphorylation data. For example, 96% of phosphosites had protein-level measurements distinguishing differential phosphorylation from altered protein abundance (Figure 1B). Proteins and phosphosites quantified in at least 50% of all deletion strains were considered for subsequent analyses (Figure 1C). Altogether, 4,475 yeast-verified open reading frames (ORFs) and 246 uncharacterized ORFs were quantified, covering 86% and 33% of all yeast verified and uncharacterized ORFs, respectively (Figure S1E).

Hierarchical clustering of all samples showed that biological duplicates clustered tightly with no batch effect from growth batches or TMT groups (Figure S1F). Figure 1D shows a small subset of the deletion strain dendrogram from Figure S1F highlighting clustering of replicates as well as no obvious grouping based on TMT batch. Figure 1E highlights some examples of known protein expression and protein phosphorylation regulation across all deletion strains. For example, GPD1 is a HOG1-dependent osmostress-induced protein and its expression is regulated by HOG pathway, which consists of HOG1, PBS2, SSK2, etc. (Albertyn et al., 1994). GPD1 showed reduced protein levels in these three kinase deletion strains. PKP1 and PKP2 are kinases that phosphorylate of PDA1 at S313 (Gey et al., 2008). PDA1 pS313 exhibited distinctly lower phosphorylation levels in strains lacking these two kinases (Figure 1E).

### Analysis of Proteomic and Phosphoproteomic Phenotypes in Deletion Strains

First, we systematically surveyed the datasets for significant protein or phosphorylation perturbations across all deletion strains at individual protein or phosphosite levels. Proteins or phosphosites quantified in ≥50% deletion strains were considered. Conservative thresholds included a standard deviation (3 × in both duplicates), and a minimum fold change difference (protein, 1.3; phosphorylation, 1.4) was used as 1% of the difference between duplicates exceeded these thresholds (Figure S2A).

**Figure 2.**
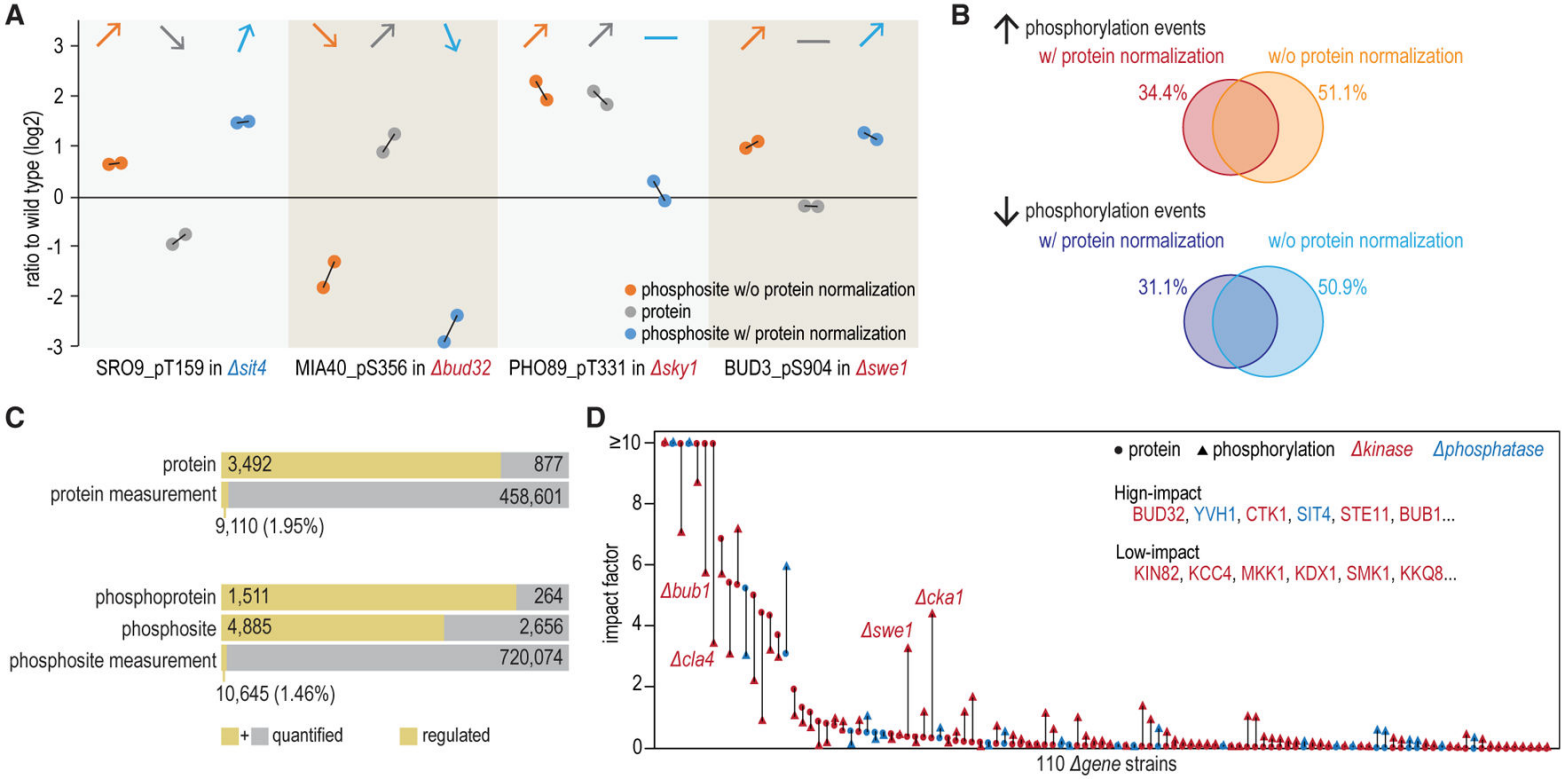
Analysis of Proteomic and Phosphoproteomic Phenotypes (A) Examples of how protein abundance alterations impacted phosphorylation quantification results. Black solid lines connect biological duplicates. (B) More than 50% of regulated phosphorylation events could be explained simply by differences at protein expression levels. Using values normalized to protein expression, many previously uncaptured phosphorylation changes were now captured. (C) Summary of phenotypes at the protein and phosphorylation levels (with protein normalization). (D) Proteome-wide and phosphoproteome-wide impact of each kinase and phosphatase. Impact factor (%) is the fraction of proteins or phosphosites affected relative to the number of quantified proteins or phosphosites. See also Figure S2.

Phosphopeptide measurements are a composite of protein expression and phosphorylation stoichiometry differences (Wu et al., 2011a). As shown in Figure 2A, SRO9 pT159 in *Δsit4* and MIA40 pS356 in *Δbud32* showed larger differences after protein abundance differences were considered. The change of PHO89 pT331 in *Δsky1* could be attributed to the protein abundance change, while the perturbation of BUD3 pS904 in *Δswe1* represented a change in phosphorylation status (Figure 2A). Overall, more than 50% of regulated phosphorylation events could be attributed to changes in protein abundance (Figure 2B). In some deletion strains, this percentage was even higher, such as 68% in *Δbud32* and 70% in *Δdbf2* (Figure S2B). With protein normalization, more than 30% of the regulated phosphorylation events were newly captured (Figure 2B). To rule out the variances from protein abundance differences and better reflect the real impact of kinase and phosphatase perturbations on phosphorylation status, we normalized the phosphorylation quantifications with protein quantifications in all subsequent data analyses unless otherwise specified.

In total, 9,110 protein-level differences and 10,645 phosphorylation-level differences were counted as regulated (Figures 2C, S3A, and S3B). Some protein kinases (BUD32, YVH1, CTK1, SIT4, etc.) exhibited high impact at both proteome and phosphoproteome levels, while others (KIN82, KCC4, MKK1, etc.) had little impact (Figures 2D and S3C). Among all regulated events, about 66% of protein measurements and 55% of phosphorylation events changed in ≥2 deletion strains (Figures S3D and S3E).

### Protein and Phosphorylation Regulation Network Analyses

**Figure 3.**
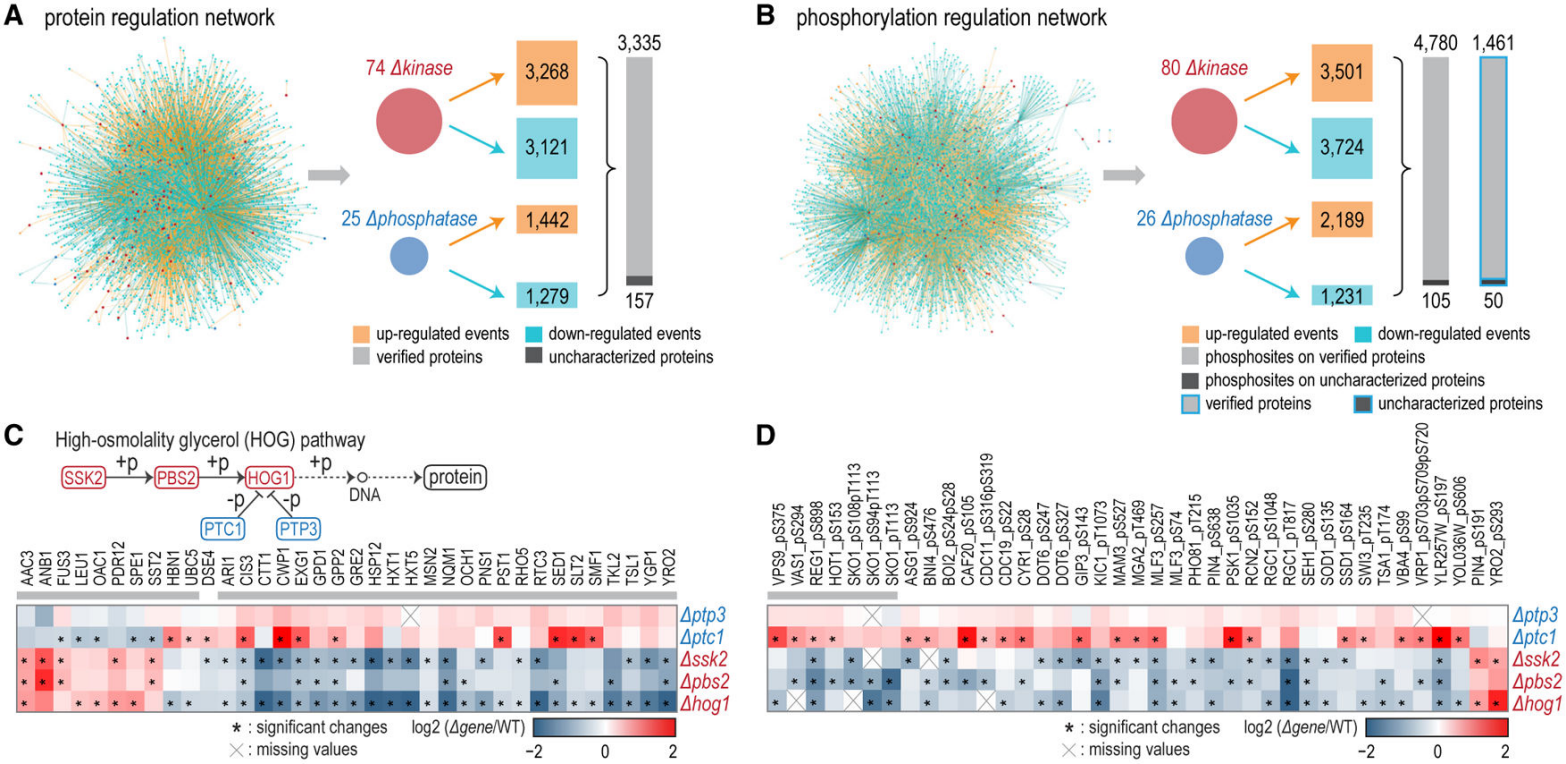
Protein Regulation Network and Phosphorylation Regulation Network Analyses (A) Protein regulation network. Overall, 4,400 downregulated and 4,710 upregulated events were captured in 74 kinase and 25 phosphatase deletion strains. (B) Phosphorylation regulation network. Together, 4,955 downregulated and 5,690 upregulated phosphorylation events were captured in 80 kinase and 26 phosphatase deletion strains. (C) Altered proteins for which cognate kinases and phosphatases were enriched in the HOG pathway (BH-adjusted p value ≤ 0.01). The gray bar above the heatmap indicates proteins showing consistent changes in previous studies. (D) Regulated phosphosites for which cognate kinases and phosphatases were enriched in the HOG pathway (BH-adjusted p value ≤ 0.01). Gray bar indicates known target phosphoproteins or phosphosites of the HOG pathway. See also Figure S3 and Tables S3 and S4.

We organized all significant protein or phosphorylation perturbations in corresponding deletion strains as regulation networks (Figures 3A and 3B; Tables S3 and S4). The protein regulation network consisted of 74 kinase and 25 phosphatase regulators, 3,492 protein effectors, 4,400 downregulations, and 4,710 upregulations. The phosphorylation regulation network was composed of 80 kinases and 26 phosphatases, 4,885 regulated phosphosites on 1,511 proteins, 4,955 downregulated events, and 5,690 upregulated events. In kinase deletion strains, downregulated phosphorylation events significantly enriched direct substrates in previous studies or NetworKIN predictions (Figure S4A) (Breitkreutz et al., 2010; Horn et al., 2014; Mok et al., 2010; Ptacek et al., 2005; Sharifpoor et al., 2011).

The HOG pathway regulates stress responses and is mainly related to the adaptation to hyperosmotic stress in yeast (Romanov et al., 2017). Components and downstream targets of the HOG pathway have been extensively characterized. First, we examined the proteomic and phosphoproteomic perturbations in strains lacking HOG pathway components to assess the datasets at the pathway level. With enrichment analysis of regulators, we found 36 proteins that were distinctly regulated by the HOG pathway (Figure 3C). Among them, 35 are known osmolality-responsive genes or showed consistent changes at mRNA level in cognate deletion strains previously (Baccarini et al., 2015; García et al., 2009; Greatrix and van Vuuren, 2006; Nadal-Ribelles et al., 2012; Norbeck and Blomberg, 1997; Norbeck et al., 1996; van Wageningen et al., 2010), demonstrating the robustness and reliability of our analysis. Similarly, we detected 39 phosphosites (on 33 proteins) that were significantly modulated by the HOG pathway (Figure 3D). Among them, SKO1 and HOT1 are target proteins of this pathway and HOT1 pS153 is one of the target phosphosites (Alepuz et al., 2003). Another study also revealed that VPS9 S375, VAS1 S294, REG1 S898, and HOG1 S153 were HOG pathway effectors (Romanov et al., 2017). Our results successfully reflected linear relationships among components of the HOG pathway cascades and indicate that our strategy was sensitive enough to analyze pathways active even at basal levels.

**Figure 4.**
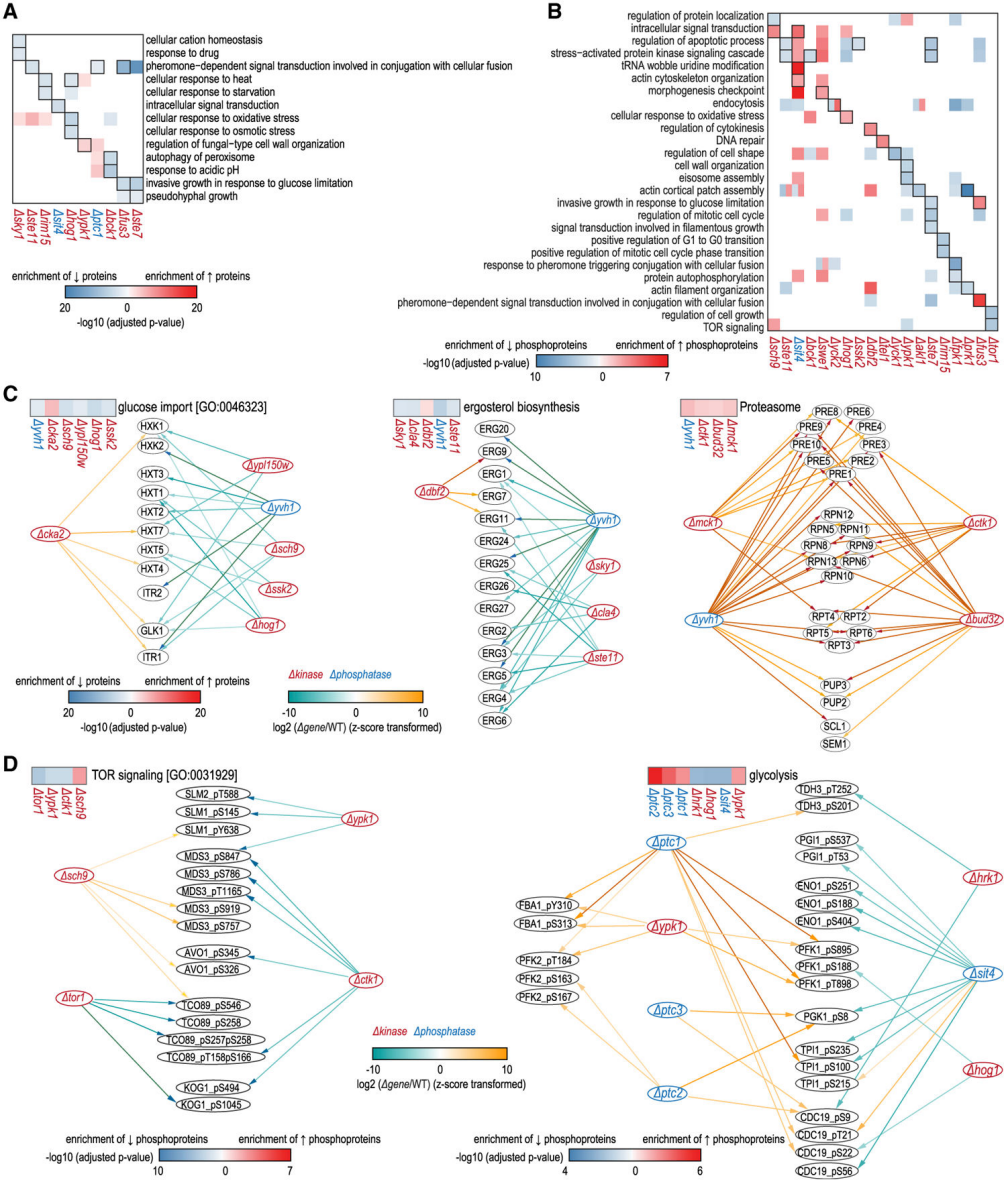
Functional Relationships by Gene Ontology (GO) Enrichment Analysis of Protein Regulation Networks and Phosphorylation Regulation Networks For each kinase and phosphatase, GO categories enriched among their regulated proteins or phosphoproteins were computed. (A) Part of the enrichment analysis results on protein effectors using the Uniprot biological pathways database (see Table S3 for full results). Cells with black borders indicate kinases and phosphatases for which known pathways were enriched in their protein effectors. (B) Part of the enrichment analysis results on phosphoprotein effectors using the Uniprot biological pathways database (see Table S4 for full results). Cells with black borders are the same as in (A). (C) Examples showed functionally related kinases and phosphatases in regulating proteins involved in glucose import, ergosterol biosynthesis, and proteasome. (D) Examples of subnetworks of kinases and phosphatases coordinately modulating the TOR signaling pathway and glycolysis. See also Figure S4 and Tables S3 and S4.

Next, with Gene Ontology enrichment analysis on protein and phosphoprotein effectors, we explored further functional relationships between kinases and/or phosphatases from biological pathway, protein complex, biochemical pathway, and cellular component perspectives. Using the Uniprot biological pathways database, at the protein level we determined that >700 pathways were significantly impacted, and 42% were modulated by ≥2 functionally related kinases and phosphatases (Table S3). Similarly, at the phosphorylation level we observed that >500 pathways were distinctly affected, and 48% were altered by ≥2 associated kinases and phosphatases (Table S4). For each active kinase or phosphatase, averages of 16 and 8 pathways were enriched in affected proteins and phosphoproteins, respectively. The effects of kinases and phosphatases on controlled biological processes were broad at both the proteome and phosphoproteome levels. For many kinases and phosphatases, related pathways were enriched among their effectors (Figures 4A and 4B). In addition, in cellular component enrichment analysis for phosphoprotein effectors, many enriched categories were known subcellular localizations of their corresponding kinases or phosphatases (Figure S4B). For instance, five cellular components were enriched in the downregulated phosphoproteins in *Δhsl1*, and four were known localizations of HSL1. This cellular component enrichment analysis can be used to predict cellular compartments where active kinases or phosphatases function.

Kinases and phosphatases that impinge on the same biological process or protein complex imply functional coordination in regulating corresponding cellular functions. As shown in Figure 4C, glucose import (GO: 0046323) proteins were concertedly modulated by five kinases and a phosphatase. It is known that the HOG pathway (SSK2 and HOG1 in this result) transcriptionally regulates hexose transporters (HXTs) (Nadal-Ribelles et al., 2012). This sub-network indicated that CKA2, SCH9, YPL150W, and YVH1 also played roles in maintaining the homeostasis of proteins involved in glucose import. Ergosterol biosynthesis enzymes were significantly decreased in *Δyvh1, Δsky1, Δcla4*, and *Δste11*, and increased in *Δdbf2*, indicating a functional sub-network of these regulators in modulating ergosterol biosynthesis (Figure 4C). The overall lower levels of ergosterol biosynthesis enzymes in *Δyvh1* could also explain the significant decreased ergosterol abundance observed in a previous study (da Silveira Dos Santos et al., 2014). Proteasome proteins all went up in *Δyvh1, Δmck1, Δctk1*, and *Δbud32*, suggesting associated roles of these kinases and phosphatases in proteasome homeostasis (Figure 4C). There is likely a connection between upregulated proteasomes and growth defects in these mutants as well, as all four of these mutants have shown decreased fitness (*Saccharomyces* Genome Database) (Cherry et al., 2012).

Phosphoprotein effectors of TOR1, SCH9, YPK1, and CTK1 were all enriched in the TOR signaling pathway (Figure 4D, left). TOR1 is a subunit of the TORC1 complex, which plays a central role in the TOR signaling pathway in response to external stimuli. SCH9 and YPK1 are downstream components of the TOR signaling pathway in yeast (Urban et al., 2007; Yerlikaya et al., 2016). This evidence supported our results that these kinases were linked via the TOR signaling pathway. The altered phosphorylation status of TOR signaling members in *Δtor1, Δsch9*, and *Δypk1* could be results of direct regulation on downstream targets or feedback regulation. Kinase YPK1, HRK1, and HOG1 and phosphatase PTC1, PTC2, PTC3, and SIT4 also showed coordinated phosphorylation regulation on the glycolysis pathway (Figure 4E, right). Kinase CLA4, TOR1, PTK2, and SKY1 and phosphatase YVH1 and PTC1 displayed a functional sub-network in modulating the phosphorylation status of the vacuolar transporter chaperone complex (Figure S4C). These example sub-networks suggested functionally related kinases and phosphatases in controlling specific biological functions via regulation of protein homeostasis or modification of phosphorylation status.

In addition to enrichment analysis, we also examined our data for potential mechanisms that could explain phenotypes observed in the literature. A previous study investigating lipidomic perturbations in yeast kinase and phosphatase mutants uncovered novel candidates for regulation of sphingolipid homeostasis (da Silveira Dos Santos et al., 2014). We extracted all significantly altered sphingolipid metabolism enzymes and their regulators from the protein and phosphorylation regulation networks (Figure S4D). Here, phosphorylation data without protein normalization were shown, as we aimed to explore connections between phosphorylated enzymes and their substrates (Oliveira et al., 2012). In our data, sphingolipid metabolism enzymes showed altered abundance or phosphorylation levels in about 30% of previously reported low-hit or high-hit deletion strains (Figure S4D). For example, in *Δyvh1* decreased ceramides were observed previously (da Silveira Dos Santos et al., 2014). In our data, three key enzymes (TSC10, LCB1, and LCB2), which control the flow of substrates (palmitoyl-CoA and serine) through the single point of entry in the ceramide synthesis pathway, were decreased in *Δyvh1*. Due to the lack of information on how phosphorylation affects the activities of sphingolipid metabolism enzymes, it is difficult to infer specific mechanisms from a phosphorylation perspective. However, our data provide a valuable resource for researchers to conduct further investigations.

In summary, in line with a previous study, our results showed that at steady state, inactivation of most kinases and phosphatases affected large parts of the phosphorylation-modulated signal transduction network (Bodenmiller et al., 2010). But not only signal transduction machinery, we found that protein homeostasis was also impacted profoundly on a broad range of biological processes. Furthermore, functionally connected kinases and phosphatases inferred from the enrichment analysis can shed light on future investigations on signaling pathway architectures (Tables S3 and S4).

### Functional Correlations through Regression Analysis of *Δgene-Δgene* Perturbation Profiles

**Figure 5.**
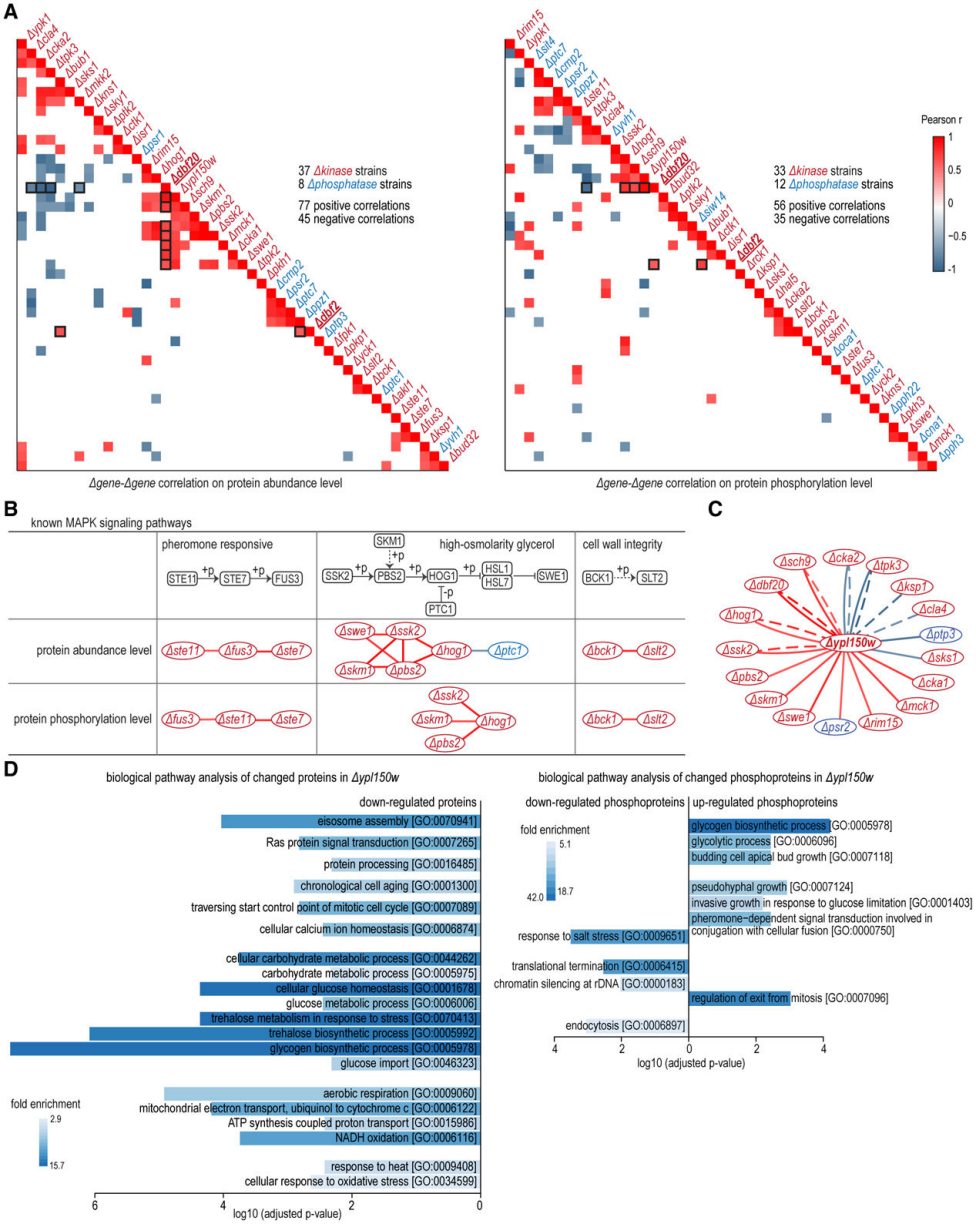
Functional Correlations through Regression Analysis on Proteomic and Phosphoproteomic Perturbation Profiles (A) Heatmaps of Pearson correlation (r) for pairs of *Δgene* proteomic (left) and phosphoproteomic (right) perturbation profiles (∣r∣ ≥ 0.6). Cells with black borders highlight correlations between *Δdbf2/Δdbf20* and other strains. (B) Regression analysis recapitulated known relationships between kinases and/or phosphatases in MAPK signaling pathways at both proteome and phosphoproteome levels. Edge colors denote correlation (r) values and are the same as in (A). (C) Uncharacterized kinase YPL150W and its correlated kinases and phosphatases. Edge colors denote correlation (r) and are the same as in (A). Solid edges and dashed edges indicate correlation at the proteome and phosphoproteome levels, respectively. (D) Biological pathways enriched in regulated proteins (left) and phosphosproteins (right) in *Δypl150w*. Benjamini-Hochberg FDR adjustment was applied to account for multiple hypothesis testing. See also Table S5.

Next, *Δgene-Δgene* correlations were computed through pairwise comparisons of global proteomic or phosphoproteomic perturbations (Figure 5A; Table S5). Overall 77 positive and 45 negative correlations were identified between 37 kinases and/or 8 phosphatases at the protein abundance level; 56 positive correlations and 35 negative correlations between 33 kinases and/or 12 phosphatases were discovered at the phosphorylation level. Deletion of kinases or phosphatases within the same pathway, such as STE11, STE7, and FUS3 in the pheromone responsive pathway and BCK1 and SLT2 in the CWI pathway, displayed highly significant correlations between each other (Figure 5B). Significant correlations came from similar or opposite perturbations of a subset of proteins or phosphosites. Correlated kinases and phosphatases suggested that they could be linearly or partially redundant components of a pathway, or components of crosstalking signaling pathways. Protein abundance is the readout that integrates effects from regulatory pathways converging on protein synthesis and/or protein degradation, while phosphorylation dynamics is the readout that could reflect potential direct mechanisms.

Kinase DBF2 and its paralog DBF20 both function during the cell cycle as mitotic exit network proteins, and DBF2 accounts for the majority of the DBF2/DBF20-associated kinase activity (Hotz et al., 2012; Toyn and Johnston, 1994). *Δdbf2* and *Δdbf20* strains are viable but deletion of both is lethal (Toyn and Johnston, 1994), suggesting some functional redundancy between these two kinases. Consistent with previous knowledge, our data revealed a greater impact in *Δdbf2* than *Δdbf20* at both the proteome and phosphoproteome levels (Figure S3A). Phosphoproteins altered in *Δdbf2* were also enriched in regulation of cytokinesis (GO: 0032465) (Figure 4B). Correlation of *Δdbf2* and *Δdbf20* at the phosphorylation level reflected their common function in cell-cycle progression and common regulation by SPO12 (Toyn and Johnston, 1993) (Figure 5A). Requirement of DBF2 and another protein kinase BUB1 in anaphase (Farr and Hoyt, 1998) was recapitulated by correlations between them at both protein and phosphorylation levels (Figure 5A). More interestingly, *Δdbf20* also presented correlations with other kinases, particularly components of the HOG pathway (PBS2, SSK2, HOG1, etc.). Activity of the cyclin-dependent kinase CDC28 is negatively modulated by MAPK pathways and inactivation of CDC28 was a critical factor in DBF2 and DBF20 functions (Hwa Lim et al., 2003), which explained the correlations between the DBF20 and HOG pathway components. Although common functions and regulations exist between DBF2 and DBF20, studies also indicated different roles for these kinases (Attner and Amon, 2012; König et al., 2010). The lack of correlations between the DBF2 and MAPK components could imply different functions or divergent mechanisms employed by DBF2. It is also likely that compensation from DBF20 in *Δdbf2* masked the correlation between DBF2 and MAPK components.

YPL150W is an uncharacterized kinase with an unknown function in yeast. In our data it was correlated (either positively or negatively) with 15 other kinases and 2 phosphatases (Figure 5C). Among them five kinases in the HOG pathway (SKM1, SSK2, PBS2, HOG1, and SWE1) showed positive correlations, suggesting YPL150W could be functionally associated with the HOG pathway. Additionally, biological pathway enrichment analysis of YPL150W effectors indicated its involvement in sugar metabolism, mitochondria function, stress responses, etc. (Figure 5D). YPL150W was recruited to cytoplasmic foci as cells entered the G_0_-like quiescent state (Shah et al., 2014). Accordingly, phosphoprotein effectors of YPL150W were enriched in pseudohyphal growth, responses to glucose limitation, and salt stress categories (Figure 5D). Our results further support a role of YPL150W in entering quiescence under stress in yeast cells. Overall, the information encapsulated with the *Δgene-Δgene* correlation networks provided extra biological insights for characterizing YPL150W functions and, more broadly, can be used to resolve pathway organizations from a different perspective.

### Protein Functions and Pathway Organizations by Molecular Covariance Network Analysis

Lastly, we analyzed the correlations across the complete protein dataset and phosphorylation dataset to collect covariant proteins and covariant phosphosites, respectively. Results were visualized as networks of proteins or phosphosites (nodes) and correlations (edges). With correlations strictly filtered with ∣r∣ ≥ 0.7 and Bonferroni-adjusted p value ≤ 0.001, permutation tests showed 0.05% and 0.44% false positives for the protein covariance and phosphosite covariance networks, respectively (Figure S5A).

**Figure 6.**
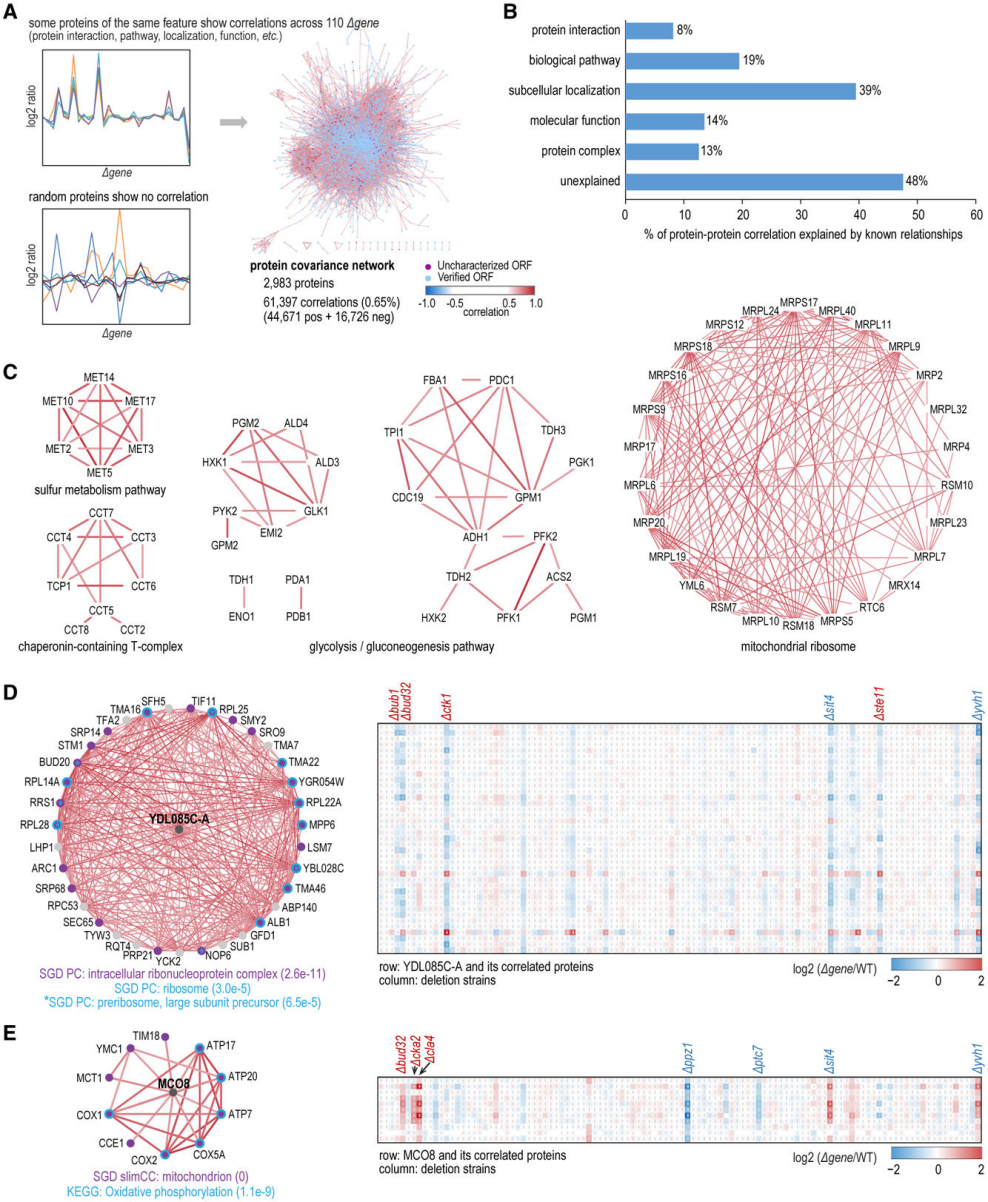
Protein Covariance Network Analysis (A) Some proteins grouped via properties (protein interaction, biological pathway, subcellular localization, molecular function, protein complex, etc.) showed consistent, changing patterns across 110 deletion strains and constituted the protein covariance network (∣r∣ ≥ 0.7, adjusted p value ≤ 0.001). (B) Percentage of protein correlations that could be explained by known biological relationships. (C) Protein covariance network analysis captured known functional structures. Edge colors denote correlation (r) and are the same as in (A). (D) Neighbor protein covariance networks uncovered functions for the uncharacterized protein YDL085C-A (left). For a given protein, neighbors were tested for GO term enrichment with Benjamini-Hochberg adjustment to account for multiple hypothesis testing. Edge colors in subnetworks on the left denote correlation (r) and are the same as in (A). Cells not labeled with a pound sign (#) in heatmaps on the right denote missing values. Heatmaps on the right show the changing patterns of YDL085C-A and its covariant proteins across all deletion strains. (E) Neighbor protein covariance networks implied functions for uncharacterized protein MCO8. Annotations are the same as in (D). See also Figures S5 and S6 and Table S6.

Overall, nearly 3,000 proteins showed >60,000 significant correlations (44,671 positive and 16,726 negative), which was 0.65% of all possible connections (Figure 6A; Table S6). About half of the protein correlations could be attributed to a particular mechanism, the highest being from shared subcellular localizations (Figure 6B). Unexplained edges may come from incomplete gene annotation and potentially represent undiscovered biology. Many known pathways or structures were captured in the protein covariance network, such as sulfur metabolism, chaperonin-containing T-complex, glycolysis/gluconeogenesis, mitochondrial ribosome, proteasome, and cytosolic ribosome (Figures 6C and S5B). Encouraged by the numbers of attributable edges and successful recapitulation of known biology, we sought to elucidate functions of uncharacterized proteins by conducting Gene Ontology enrichment analysis on their network neighbors. Many uncharacterized proteins were linked to neighbors of known functions, providing foundations for generating hypotheses about their functions (Figures 6D, 6E, and S6; Table S6). For instance, uncharacterized protein YDL085C-A was localized to the nucleus in a previous large-scale study (Huh et al., 2003). In our data, its positively correlated neighbors were significantly enriched in intracellular ribonucleoprotein complex and ribosome, suggesting a role for YDL085C-A in these structures (Figure 6D, left). A view of variance of YDL085C-A and its neighbors across all deletion strains indicated that BUB1, BUD32, CTK1, and SIT4 played associated roles in regulating the protein homeostasis of the YDL085C-A subnetwork (Figure 6D, right). As another example, MCO8 was recently named and assigned to mitochondrion with functions still unknown (Morgenstern et al., 2017). In agreement with the previous study, MCO8 was predicated to be a mitochondrial protein (Figure 6E, left). More significantly, its covariant proteins indicated a function of MCO8 in oxidative phosphorylation (Figure 6E, left). A heatmap illustration showed that the MCO8 subnetwork was coordinately modulated by BUD32, CKA2, CLA4, and PPZ1 (Figure 6E, right).

**Figure 7.**
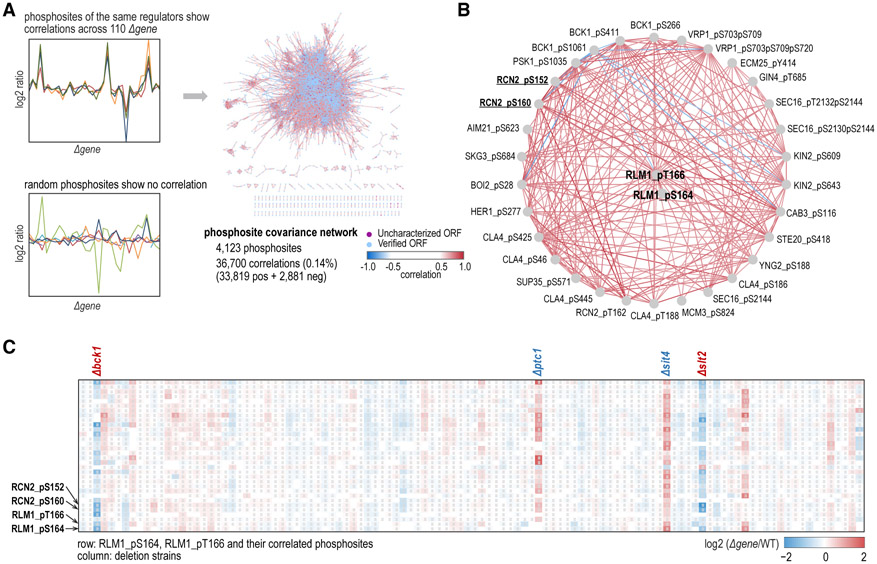
Phosphosite Covariance Network Analysis (A) Some phosphosites showed correlations across 110 deletion strains and composed the phosphosite covariance network (∣r∣ ≥ 0.7, adjusted p value ≤ 0.001). (B) Correlation between RLM1 pT166, RLM1 pS164, and their covariant phosphosites. Edge colors denote correlation (r) and are the same as in (A). (C) Changing patterns for RLM1 pT166, RLM1 pS164 and their correlated phosphosites across all deletion strains. Cells not labeled with a pound sign (#) denote missing values. See Figure S7 and Table S7.

In the phosphosite covariance network, >4,000 phosphosites displayed >36,000 distinct correlations (33,819 positive and 2,881 negative), accounting for 0.14% of all possible links (Figure 7A; Table S7). About 4.5% of the correlations were between phosphosites on the same protein. Due to the lack of functional relevance for the majority of these phosphosites, performing a similar analysis on the phosphosite covariance network is challenging. However, for a given phosphosite, its nearest-neighbor network can still reveal valuable insights on the pathway architecture in the subnetwork (Figures 7B, 7C, and S7). For example, 30 phosphosites showed similar or opposite changing patterns with RLM1 pT166, and RLM1 pS164 across all deletion strains. The heatmap of their changes across all deletion strains implied that BCK1, PTC1, SIT4, and SLT2 were involved in the dynamics of these phosphosites. Kinases BCK1 and SLT2 are known MAPK components of the CWI pathway. Phosphatase PTC1 is associated with the CWI pathway by dephosphorylating MKK1 (Tatjeret al., 2016). Phosphatase SIT4 is essential for downregulation of PKC1 activity and consequently required for the CWI pathway (Angeles de la Torre-Ruiz et al., 2002). These kinases and phosphatases all play roles in the CWI pathway and we successfully captured their functional relationships from the neighbor phosphosite covariance subnetwork of RLM1 pT166 and RLM1 pS164. RLM1 S427 and T439 are targets of SLT2 (Jung et al., 2002; Watanabe et al., 1997) and we did not identify these two phosphosites. The functions of RLM1 pT166 and RLM1 pS164 remain unknown, and they could be targets of the CWI pathway. Similarly, other phosphosites in this subnetwork also indicate specific downstream substrates of the CWI pathway. Among them, Rcn2 S152 and Rcn2 S160 are known direct substrates of SLT2 (Alonso-Rodríguez et al., 2016).

As another example, the phosphorylated DIG1 and DIG2 subnetwork also recapitulated known target proteins (BNI5, FAR1, PBS2, STE12, and STE50) and the architecture of the pheromone responsive pathway (STE7, STE11, and FUS3) (Cappell and Dohlman, 2011; Juanes and Piatti, 2016; Kanehisa and Goto, 2000) (Figure S7A). In a similar way, potential novel pathway architectures could be inferred from the phosphosite covariance network. For instance, the HIF1 pS353 subnetwork suggested a functional module of BUB1, BUD32, CTK1, DBF2, and DUN1 (Figures S7B-S7E). Overall, for a given phosphosite, the phosphosite covariance network is a useful resource for retrieving information of co-regulated phosphosites and the regulatory mechanism employed, which can help to illuminate new pathway architectures.

## DISCUSSION

With the SL-TMT strategy, measurements of both protein expression and phosphorylation levels occur on the same labeled starting material. This minimizes differences due to sample handling and labeling effects while maintaining excellent depth of analysis (>4,000 proteins across all samples). We present here a systems-level analysis of responses to interventions of phosphorylation signaling pathways with both proteomic and phosphoproteomic readouts in yeast. Our datasets showed high agreement with preexisting results. More significantly, they have predictive power, which will help us to understand and model phosphorylation signaling transduction networks in yeast.

The numbers of proteins and phophosites with altered levels caused by the ablation of a specific kinase or phosphatase varied considerably. More than 50% of the deletion strains exhibited a modest impact at protein abundance and phosphorylation levels (Figure S3C). The lack of a strong proteomic or phosphoproteomic phenotypes may be caused by the absence or inactivation of kinases or phosphatases under non-inducing growth conditions, or compensation or buffering effects from functionally redundant proteins (van Wageningen et al., 2010). For instance, single deletion of paralog genes would have minimal effect on the proteome and phosphoproteome if their substrates are overlapping, such as *Δrck1/Δrck2, Δpkh1/Δpkh2, Δnpr1/Δprr2*, and *Δmkk1/Δmkk2* in this work. Various environmental or pharmacological conditions, such as different culture conditions, different carbon sources, or interventions with activators, can be used to further explore functions of silent kinases and phosphatases. For example, Zelezniak et al. (2018) observed profound and broader impacts on the metabolic enzyme proteome in 97 kinase deletion strains grown in minimal media.

The modulation of the homeostasis of proteins involved in a specific function could be achieved by coordinated regulation of the phosphorylation status of common transcription factors. For instance, in proteasome homeostasis regulation (Figure 4C), we found that in total 444 phosphorylation events (84 unique phosphosites on 34 transcription factors) went up in *Δmck1, Δyvh1, Δctk1*, and *Δbud32*. According to the Yeastract database (Teixeira et al., 2018), each of these 34 transcription factors targeted at least one of the 26 upregulated proteasome proteins in Figure 4C. Former study also revealed consistent mRNA changing patterns of these 26 proteins in *Δmck1, Δyvh1*, and *Δctk1* (van Wageningen et al., 2010). Under the assumption that these transcription factors are activated by phosphorylation, this result implied that the proteasome protein augmentations in *Δmck1, Δyvh1, Δctk1*, and *Δbud32* were mediated by increased phosphorylated transcription factors.

In *Δgene-Δgene* correlation network analysis, similar profiles of deletion strains indicated common downstream effects between kinases and/or phosphatases. Functions or pathways with which a specific kinase or phosphatase are involved can be inferred from neighbors in the *Δgene-Δgene* correlation network. Groups of covariant substrates were revealed and their changing patterns across kinases and phosphatases could imply novel functional organizations of signal pathways. It should be noted that some correlations or structures could be missed due to the inactivation or absence of a particular kinase and/or phosphatase.

This work measured both the global proteome and phosphoproteome, and further investigations of phosphorylation stoichiometries can be conducted for some phosphosites (Olsen et al., 2010; Wu et al., 2011b). However, caution is advised for the interpretation of the stoichiometry results, as some methods have special requirements for the data in order to return valid occupancies. Additionally, the data in this work can also be used to facilitate the development of new methods for stoichiometry calculation.

## STAR★METHODS

### LEAD CONTACT AND MATERIALS AVAILABILITY

Further information and requests for resources and reagents should be directed to and will be fulfilled by the Lead Contact, Steven P. Gygi (steven_gygi@hms.harvard.edu). This study did not generate new unique reagents.

### EXPERIMENT MODEL AND SUBJECT DETAILS

The parental wild-type *Saccharomyces cerevisiae* strain for this study was the haploid MATalpha BY4742. Single gene deletion derivatives of BY4742 were obtained through the gene deletion consortium (Giaever et al., 2002). All gene deletions were confirmed by either proteomics or PCR assays. Single lots of YPD media and YPD+G418 (200 μg/mL) plates were used. Yeast from a −80°C stock were streaked onto YPD+G418 plates and incubated (30°C, ~48 h). Starter cultures (3 mL YPD media) were inoculated with a patch of yeast cells and incubated overnight (30°C, 230 rpm). YPD media (15 ml) were inoculated with starting OD_600nm_ = 0.1, incubated (30°C, 230 rpm) and harvested at OD_600nm_≈1.0. Cells were washed 3 times with cold water and pelleted by centrifugation (4000 *g*, 5 min, and 4°C). The pellets were stored at −80°C. All deletion strains were grown in 16 batches. WT cultures were grown under exactly the same condition as deletion strains.

### METHOD DETAILS

#### Sample Preparation Based on a Streamlined Tandem Mass Tag (SL-TMT) Protocol

Sample preparation followed a previously published protocol with minor adjustments (Navarrete-Perea et al., 2018). Briefly, yeast pellets were lysed by bead-beating in lysis buffer (8 M Urea, 200 mM HEPES, pH 8.5) supplemented with protease inhibitors and phosphatase inhibitors. Protein concentration was determined with BCA assays. Samples were reduced with 5 mM TCEP, alkylated with 10 mM iodoacetamide and then quenched with 10mM DTT. For each sample, a total of 100 μg protein was chloroform-methanol precipitated and reconstituted in 100 μL 200 mM HEPES (pH 8.5). Samples were digested by Lys-C overnight at room temperature and then trypsin for 6 h at 37°C, both at 1:100 protease-to-protein ratio. TMT11-plex reagents were reconstituted according to manufacturer’s instructions and then diluted 4 times with acetonitrile. To each digest 40 μL TMT reagent was added for labeling. Each deletion strain was randomly assigned into one of 14 TMT groups. Duplicates of each group increased the number of TMT groups to 28. To check labeling efficiency, 2 μL of each sample was pooled, desalted and analyzed by MS. After labeling efficiency check, samples were quenched by adding 9 μL 5% hydroxylamine. All samples were subsequently pooled into 28 TMT groups with a wild-type in each and desalted with 100 mg Sep-Pak solid-phase extraction columns. Pierce High-Select Fe-NTA phosphopeptide enrichment kit was used to enrich phosphopeptides from the pooled mixture. Unbound fractions were desalted and then fractionated with basic-pH reversed-phase high-performance liquid chromatography. Fractions were collected in a 96-well plate and combined for a total of 12 fractions prior to desalting and subsequent LC-MS/MS analysis.

#### MS Analysis

Proteomic data were collected on an Orbitrap Fusion mass spectrometer (ThermoFisher Scientific) coupled to a Proxeon EASY-nLC 1000 liquid chromatography (LC) pump (ThermoFisher Scientific). Peptides were separated on a 35 cm column (i.d. 100 μm, Accucore, 2.6 μm, 150 Å) packed in-house using a 120 min linear gradient from 2% to 23% and a subsequent 15 min linear gradient from 23% to 36% of acetonitrile with 0.1% formic acid at 550 nl/min. MS1 data were collected using the Orbitrap mass analyzer (120,000 resolution at 200 m/z; 350-1400 m/z; maximum injection time 50 ms; AGC 4e5). Determined charge states between 2 and 5 were required for sequencing and a 120 s dynamic exclusion window was used. Data-dependent “Top10” MS2 scans were performed in the ion trap with CID fragmentation (isolation window 0.7 Da; Turbo; 400-2000 m/z; NCE 35%; maximum injection time 120 ms; AGC 1e4). MS3 quantification scans were performed using multi-notch MS3-based TMT method (10 notches; 50,000 resolution at 200 m/z; NCE 65%; maximum injection time 150 ms; AGC 1.5e5) (McAlister et al., 2014). All data were collected in positive ion mode and were centroided online.

Phosphoproteomic samples were injected twice on an Orbitrap Lumos mass spectrometer (ThermoFisher Scientific) coupled to a Proxeon EASY-nLC 1200 liquid chromatography (LC) pump (ThermoFisher Scientific). For analysis without multistage activation, phosphopeptides were separated on a 35 cm column (i.d. 100 μm) packed in-house with reversed-phase materials (Accucore, 2.6 μm, 150 Å) using a 90 min linear gradient from 3% to 10%, a subsequent 65 min linear gradient from 10% to 18% and a final 10 min linear gradient from 18% to 28% of acetonitrile with 0.1% formic acid at 550 nl/min. For analysis with multistage activation, phosphopeptides were separated on the same column using a 90 min linear gradient from 5% to 16%, a subsequent 65 min linear gradient from 16% to 22% and a final 10 min linear gradient from 22% to 30% of acetonitrile with 0.1% formic acid at 450 nl/min. MS1 data were collected using an Orbitrap mass analyzer (120,000 resolution at 200 m/z; 350-1400 m/z; maximum injection time 50 ms; AGC 1e6). Determined charge states between 2 and 5 were required for sequencing and a 120 s dynamic exclusion window was used. Data-dependent “top 10” MS2 scans were performed in the ion trap with CID fragmentation with or without multistage activation for two injections (Turbo; 400-2000 m/z; NCE 35%; maximum injection time 120 ms; AGC 1e4). MS3 quantification scans were performed using multi-notch MS3-based TMT method (10 SPS ions; 50,000 resolution at 200 m/z; NCE 65%; maximum injection time 250 ms; AGC 1.5e5). All data were collected in positive ion mode and were centroided online.

### QUANTIFICATION AND STATISTICAL ANALYSIS

#### MS Data Analysis

MS data were analyzed with in-house software. Raw files were initially converted to mzXML for processing. Database searching included all entries from the *Saccharomyces* Genome Database (SGD, 2014). This database was concatenated with one composed of all protein sequences in reversed order. Searches were performed using a 50 ppm precursor ion tolerance and 0.9 Da product ion tolerance. The wide mass-tolerance window for precursors was chosen to maximize sensitivity in conjunction with SEQUEST searches and linear discriminant analysis (Huttlin et al., 2010). TMT tags on lysine residues and peptide N termini (+229.1629 Da) and carbamidomethylation of cysteine residues (+57.0215 Da) were set as static modifications, while oxidation of methionine residues (+15.9949 Da) was set as a variable modification. For phosphoprotein analysis, +79.9663 Da was set as a variable modification on serine, threonine, and tyrosine residues.

Peptide-spectrum matches (PSMs) were adjusted to a 1% false discovery rate (FDR) (Elias and Gygi, 2007, 2010). PSM filtering was performed using a linear discriminant analysis as described previously (Huttlin et al., 2010), while considering the following parameters: XCorr, ΔCn, missed cleavages, peptide length, charge state, and precursor mass accuracy. Each run was filtered separately. Protein-level FDR was subsequently estimated. For each protein across all samples, the posterior probabilities reported by the LDA model for each peptide were multiplied to give a protein-level probability estimate. Using the Picked FDR method (Savitski et al., 2015) proteins were filtered to the target 1% FDR level.

Phosphorylation site localization was determined using AScore algorithm (Beausoleil et al., 2006). AScore is a probability-based approach for high-throughput protein phosphorylation site localization. Specifically, a threshold of 13 corresponded to 95% confidence in site localization.

For TMT reporter ion quantification, a 0.003 Da window around the theoretical m/z of each reporter ion was scanned, and the nearest m/z was used. Reporter ion intensities were adjusted to correct for the isotopic impurities of the different TMT reagents according to manufacturer specifications. Peptides were filtered for a summed signal-to-noise of 200 across all 11 TMT channels and an isolation specificity of at least 0.5 in the MS1 isolation window. For each protein, the filtered unique peptide TMT values were summed to create non-normalized protein quantifications.

To control for differential protein loading within an 11-plex, the summed protein quantities were adjusted to be equal within an 11-plex. Phosphosite quantifications were also normalized by correction factors generated in this process to account for protein loading variance. Following this, values were log2-transformed, and within each 11-plex the bridge channel (wild-type strain) protein or phosphosite quantity was subtracted from each sample quantity to create a ratio to the wild-type. For each protein and phophosite, there is some measurement error in the measurement of the bridge sample. To account for this, within each 11-plex, the trimmed mean protein or phosphosite expression was centered at 0. Finally, 11-plexes were joined by protein or phosphosite identification to create the complete datasets. Phosphosite quantifications were further normalized by cognate protein ratios when needed.

#### Identification of Proteomic and Phosphoproteomic Phenotypes in Δgene Strains

Proteomic and phosphoproteomic phenotypes were determined at individual protein or phosphosite level. Proteins or phosphosites quantified in at least 50% of all deletion strains were considered in this analysis. For each protein or phosphosite, duplicates were merged and then a trimmed standard deviation (SD) was calculated with top 5% and bottom 5% of changes removed. For proteins or phosphosites quantified in both biological duplicates and one of the duplicates, 3 SD cutoff and 6 SD cutoff were applied, respectively. Proteins and phosphosites were further filtered with log2-ratio cutoffs 0.38 and 0.5, respectively. Of all delta log2-ratios between biological replicates, 1.2% and 0.9% showed values higher than 0.38 and 0.5 for protein data and phosphosite data, respectively. Results were visualized as regulation networks with Cytoscape 3.6.0 (Shannon et al., 2003). Analysis was conducted in R 3.4.2.

#### Regression Analysis of Δgene-Δgene Perturbation Profiles

For pairwise combinations of all deletion strains, linear regression analysis was conducted at protein and phosphorylation levels, respectively. Fold changes of deleted proteins in cognate strains were set as missing values to avoid false correlations caused by gene deletions. Log2-ratios ≥ 0.38 or ≥ 0.5 were used for protein and phosphosite data, respectively. A minimum of 25 proteins or phosphosites were required. These measurements were fit to a line and the associated Pearson correlation coefficient (r) was reported. A ∣r∣ ≥ 0.6 cutoff was applied and maximum Benjamini-Hochberg adjusted p values were 0.003 for results from proteomic and phosphoproteomic profiles. For pairs of deletion strains showing low correlations or lacking a sufficient number of proteins or phosphosites that met the aforementioned criteria, the Pearson coefficient was reported as 0. Results were visualized with hierarchical clustering with R package “pheatmap” 1.0.8 and Cytoscape 3.6.0. Analysis was conducted in R 3.4.2.

#### Protein Covariance Network and Phosphosite Covariance Network Analysis

For all pairwise combination of proteins or phosphosites, regression analysis was performed using log2-ratios having measurements in at least 50% of all *Δgene* strains in the pair. Fold changes of deleted proteins in corresponding strains were set as missing values to avoid false covariance caused by gene deletions. Pearson regression analysis was conducted to obtain correlation coefficients (r). All p values were corrected for multiple hypothesis testing (Bonferroni) and correlations where ∣r∣ ≥ 0.7 and adjusted p value ≤ 0.001 were reported. Permutation tests (n = 1000) were conducted to estimate false positives. Covariance networks were visualized with Cytoscape 3.6.0. Percentages of protein-protein correlations explained by known relationships were computed by matching BioGRID (downloaded on April 23, 2018) (Stark et al., 2006) for protein interactions, SGD GO slim pathway database for biological pathways, SGD GO slim cellular component database for subcellular localizations, SGD GO slim function database for molecular functions and SGD protein complex database (downloaded on August 27, 2018) (Cherry et al., 2012) for protein complexes. Analysis was conducted in R 3.4.2.

#### Gene Ontology (GO) Enrichment Analysis

For enrichment analysis of regulators (kinases and phosphatases) against HOG pathway components, 6 kinases and phosphatases in HOG pathway were tested, including SSK22, SSK2, PBS2, HOG1, PTC1 and PTP3. These six kinases and phosphatases showed impact at protein or phosphorylation levels and were within three cascades of HOG1 according to KEGG pathway database (downloaded on September 19, 2018) (Kanehisa and Goto, 2000). All 99 kinases and phosphatases showing effect at protein level, or 106 kinases and phosphatases displaying impact at phosphorylation level were used as background for protein data and phosphorylation data, respectively. Enrichment was computed via hypergeometric distribution tests and Benjamini-Hochberg adjusted p values were filtered at 1%. Two proteins and four phosphosites showing inconsistent altered patterns in HOG pathway were further removed.

For enrichment analysis of effectors (regulated proteins or phosphoproteins), databases used included KEGG yeast pathway database (downloaded on September 19, 2018) (Kanehisa and Goto, 2000), Uniprot yeast biological pathway database (downloaded on August 21, 2018) (UniProt Consortium, 2019), SGD biochemical pathway database, SGD protein complex and SGD GO slim cellular component databases (downloaded on August 27, 2018) (Cherry et al., 2012). Multiple phosphosites on a phosphoprotein were counted as one. All perturbed proteins or phosphoproteins in regulation networks were used as background. Enrichment was calculated via hypergeometric distribution tests for upregulated proteins and downregulated proteins separately. P values were corrected across all deletion strains and all GO categories per database with Benjamini-Hochberg method and then filtered at 1%.

For enrichment analysis of covariant proteins, the databases used were KEGG yeast pathway database (downloaded on September 19, 2018) (Kanehisa and Goto, 2000), Uniprot yeast biological pathway database (downloaded on August 21, 2018) (UniProt Consortium, 2019), SGD protein complex and SGD GO slim cellular component databases (downloaded on August 27, 2018) (Cherry et al., 2012). For a given protein, all positively correlated neighbors were tested. All proteins (except for the one surveyed) having positive correlations with others were used as background. Enrichment was calculated via hypergeometric distribution tests. P values were corrected across all nodes and all GO categories per database with Benjamini-Hochberg method and then filtered at 1%.

All GO enrichment analysis was conducted in R 3.4.2.

### DATA AND CODE AVAILABILITY

The mass spectrometry data have been deposited to the ProteomeXchange Consortium with the dataset identifier PXD015575.

## Supplementary Material

Table S5

Table S3

Table S4

Tale S7

Table S1

Document S1

Table S6

Table S2

## Figures and Tables

**Table:** KEY RESOURCES TABLE

REAGENT or RESOURCE	SOURCE	IDENTIFIER
Experimental models: organisms/strains		
*Saccharomyces cerevisiae* kinase/phosphatase deletion strains	Giaever et al., 2002	http://www-sequence.stanford.edu/group/yeast_deletion_project/deletions3.html
Chemicals, Peptides, and Recombinant Proteins		
YPD medium	Research Product International	Cat. # Y20090-5000.0
Protease inhibitors	Roche	Cat. # 11836170001
Phosphatase inhibitors	Roche	Cat. # 04906837001
Pierce BCA Protein Assay Kit	ThermoFisher	Cat. # 23225
TMT 11-plex reagents	ThermoFisher	Cat. # 90406, Cat. # A34807
Sep-Pak solid-phase extraction column	Waters	Cat. # WAT036820
High-Select Fe-NTA phosphopeptide enrichment kit	ThermoFisher	Cat. # A32992
Deposited Data		
Mass spectrometry data	This paper	ProteomeXchange (PXD015575)
Software and Algorithms		
In-house mass spectrometry data analysis software	Huttlin et al., 2010	N/A
Cytoscape 3.6.0	Shannon et al., 2003	https://cytoscape.org/
R 3.4.2	N/A	https://www.r-project.org/
R package “dplyr” 0.7.4	N/A	https://cran.r-project.org/web/packages/dplyr/index.html
R package “pheatmap” 1.0.8	N/A	https://cran.r-project.org/web/packages/pheatmap/index.html
R package “tidyr” 0.8.0	N/A	https://cran.r-project.org/web/packages/tidyr/index.html
R package “tibble” 1.4.2	N/A	https://cran.r-project.org/web/packages/tibble/index.html
Other		
Uniprot biological pathway database	UniProt Consortium, 2019	https://www.uniprot.org/
KEGG pathway database	Kanehisa and Goto, 2000	https://www.genome.jp/kegg/
BioGRID protein-protein interaction database	Stark et al., 2006	https://thebiogrid.org/
SGD GO database and protein complex database	Cherry et al., 2012	https://www.yeastgenome.org/

## Highlights

- SL-TMT strategy enables (phospho)proteome analysis of 110 yeast deletion strains
- Complete interpretation of phosphorylation data requires protein normalization
- Kinase and phosphatase relationships are characterized at multiple levels
- Network analyses illuminate phosphorylation signaling pathway organizations
