# Supplementary material for: Investigation of Proteomic and Phosphoproteomic Responses to Signaling Network Perturbations Reveals Functional Pathway Organizations in Yeast
Source: Cell Rep. Author manuscript; Available in PMC 2020 Jul 26. (PMC7382779; doi:10.1016/j.celrep.2019.10.034)
Supplement: Document S1 [file NIHMS1605463-supplement-Document_S1.pdf]

Cell Reports, Volume *29*

Supplemental Information

Investigation of Proteomic and Phosphoproteomic Responses to Signaling Network Perturbations Reveals Functional Pathway Organizations in Yeast

Jiaming Li, Joao A. Paulo, David P. Nusinow, Edward L. Huttlin, and Steven P. Gygi

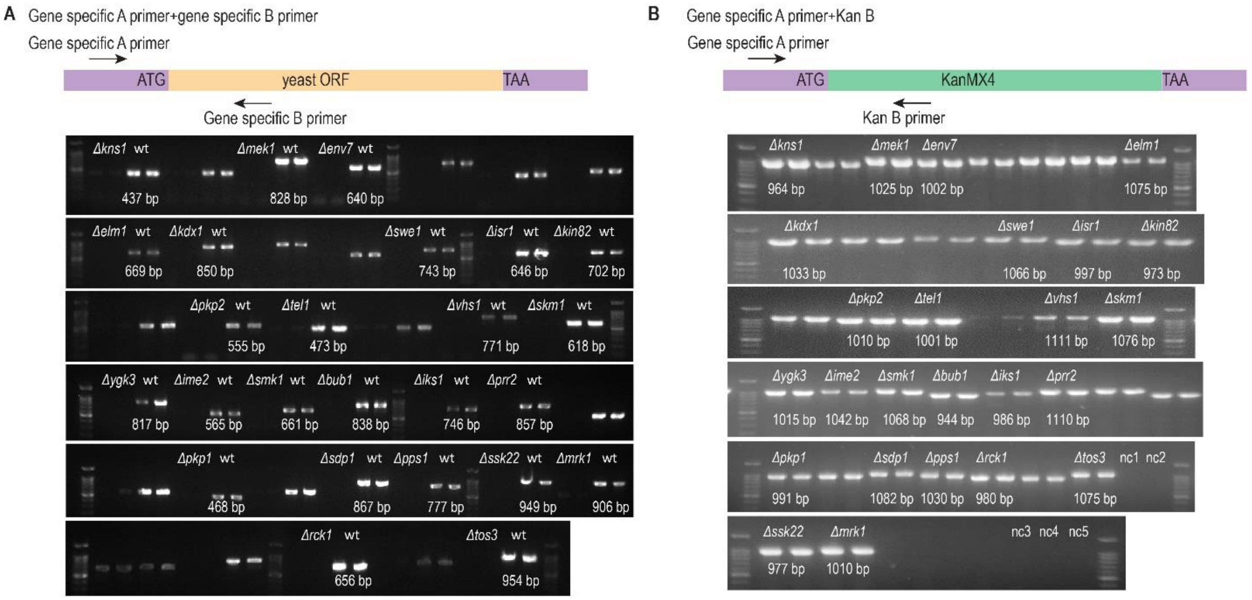

C

Proteome data	
# RAW files	336
# MS2 spectra	8,246,421
# MS3 spectra	8,246,321
# PSMs	2,071,285
# Unique peptides	88,154
# Proteins	4,647

D

Phosphoproteome data	
# RAW files	56
# MS2 spectra	1,669,147
# MS3 spectra	1,669,145
# PSMs	572,651
# Phospho PSMs	429,414
# Phosphosites	15,191

E

	# in database	Proteome	Phosphoproteome	Proteome+Phosphoproteome
# Verified ORF	5,175	4,403	2,183	4,475 (86%)
# Uncharacterized ORF	739	237	78	246 (33%)
# Dubious ORF	689	1	1	2
# Merged ORF	1			
# Blocked_reading_frame	6			
# Transposable gene	91	6	8	13
# Pseudogene	16		1	1

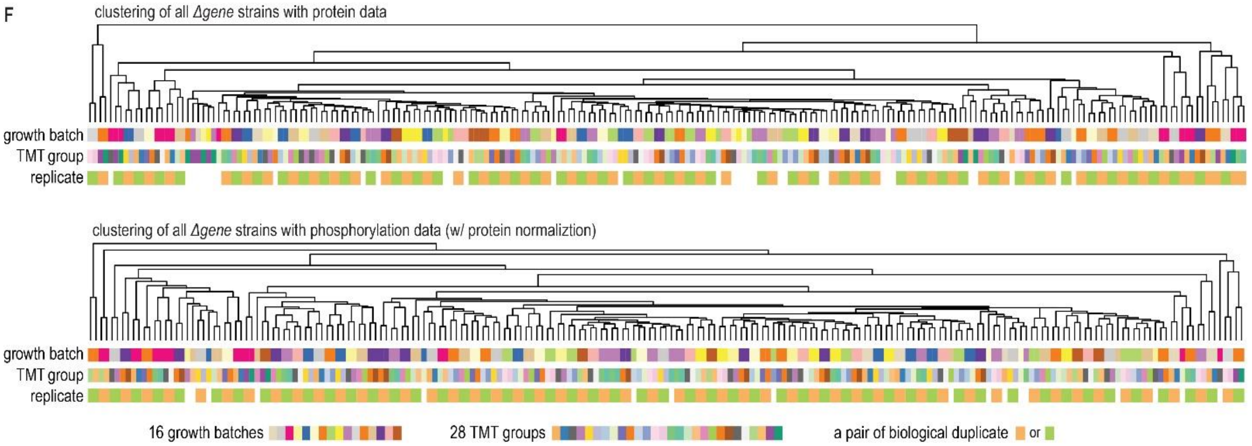

Figure S1. Data quality assessment and LC-MS/MS analysis metrics. Related to Figure 1. (A, B) PCR assay results. Gene deletions were confirmed by PCR assays if encoded proteins were not identified in proteomic data. Each of the duplicate culture was tested. "nc" in (B) indicates negative controls. **(C, D)** Numbers of Raw files, MS2 spectra, identified proteins and phosphosites, etc. in proteomic and phosphoproteomic data, respectively. **(E)** Numbers of verified ORF, uncharacterized ORF, etc. identified in the data. A high coverage of yeast proteome was captured. **(F)** Hierarchical clustering analysis of all deletion strains. Biological duplicates clustered tightly with no batch effect from growth batches or TMT groups.

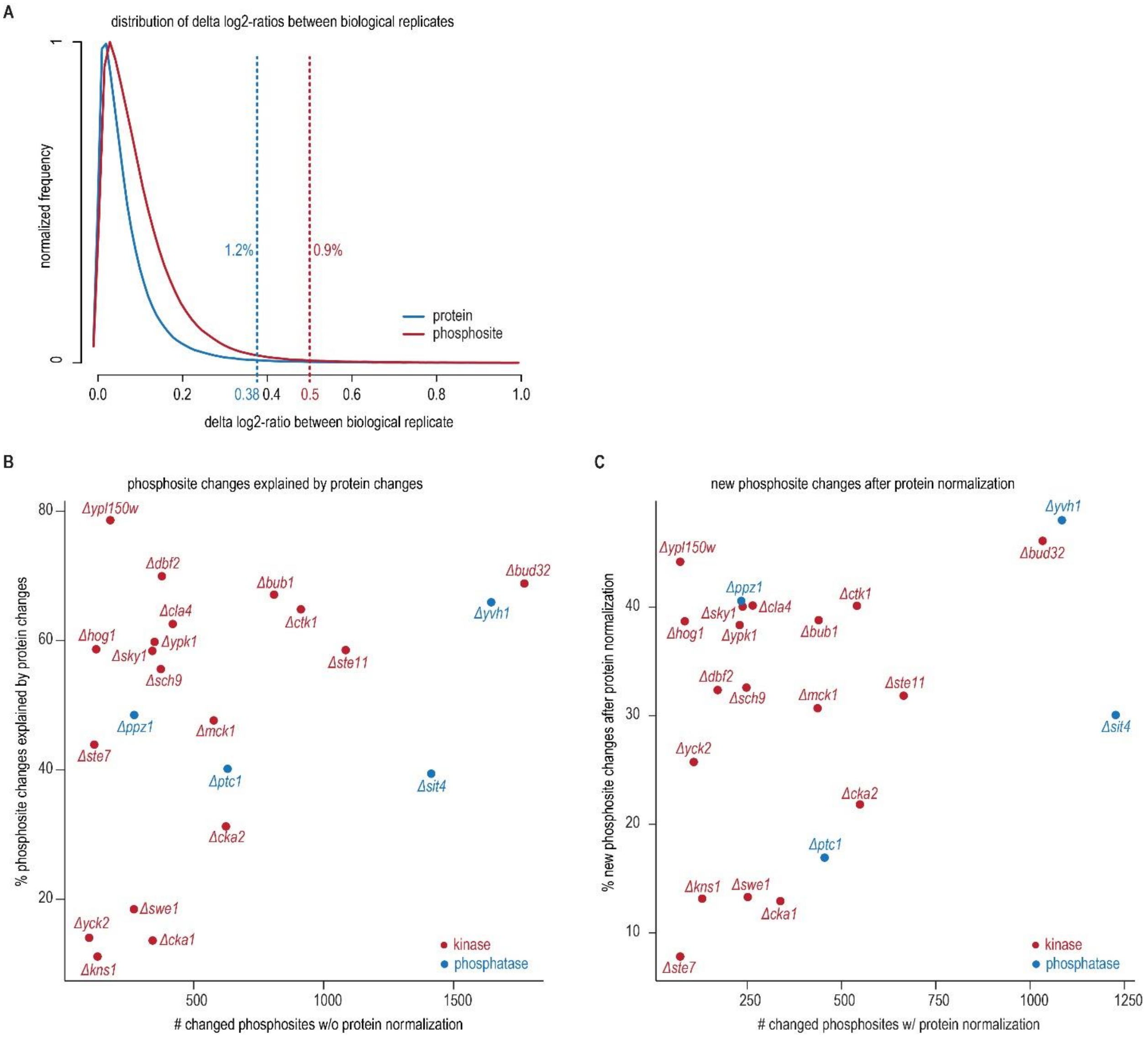

Figure S2. Evaluation of fold change thresholds and necessities of normalizing phosphorylation data with protein quantifications. Related to Figure 2. (A) Of all delta log2-ratios between biological replicates, 1.2% and 0.9% showed values higher than 0.38 and 0.5 for protein data and phosphosite data, respectively. **(B)** Percentages of changed phosphosites explained by protein differences. Deletion strains showing more than 100 perturbations are shown here. **(C)** Percentages of new phosphosite changes after protein normalization. Deletion strains showing more than 100 perturbations are included.

A

Kinase deletion	Protein up	Protein down	Phosphosite up	Phosphosite down
Δbud32	772	874	586	438
Δctk1	240	626	368	166
Δste11	258	328	173	485
Δbub1	415	171	371	62
Δcla4	398	123	189	68
Δmck1	211	90	276	154
Δsky1	146	92	125	106
Δcka2	151	84	203	339
Δdbf2	185	35	104	61
Δypl150w	28	167	25	41
Δsch9	78	113	173	68
Δypk1	99	64	137	86
Δhog1	13	72	21	57
Δrim15	28	31	11	49
Δptk2	41	11	24	23
Δdbf20	22	17	3	1
Δisr1	5	31	5	6
Δssk2	6	27	10	61
Δfus3	1	24	10	53
Δste7	2	22	6	60
Δtpk3	16	5	9	10
Δpbs2	7	11	0	31
Δskm1	5	12	1	10
Δswe1	4	13	216	28
Δbck1	9	7	45	42
Δcka1	10	5	60	271
Δksp1	9	6	6	4
Δenv7	8	3	10	27
Δdun1	9	1	74	13
Δkns1	3	6	8	116
Δmkk2	7	2	1	0
Δhal5	6	1	40	11
Δypk3	5	1	3	16
Δmrk1	6	0	1	5
Δpkh2	3	3	3	3
Δhsl1	4	2	14	15
Δfpk1	3	2	5	39
Δslt2	3	2	31	53
Δkin1	2	2	1	26
Δvhs1	2	2	2	13
Δhrk1	0	4	13	59
Δrtk1	3	1	4	11
Δmek1	1	3	0	2
Δiks1	1	3	7	0
Δtda1	3	0	0	9
Δprr1	3	0	0	7
Δsat4	2	1	0	1
Δtpk2	0	3	5	63
Δyck2	3	0	17	85
Δnpr1	3	0	2	6
Δsks1	3	0	4	13
Δssk22	1	2	4	4
Δtos3	3	0	5	6
Δpkh3	0	3	25	12
Δime2	0	3	1	0
Δalk1	2	0	0	1
Δcmk2	2	0	1	75
Δkss1	1	1	0	10
Δyck1	1	1	4	70
Δpsk2	1	1	2	13
Δprk1	2	0	3	17
Δpkh1	0	2	1	4
Δrck1	2	0	1	2
Δtel1	2	0	5	9
Δygk3	2	0	4	6
Δtor1	1	1	10	11
Δelm1	1	1	1	1
Δpkp1	2	0	20	5
Δrim11	1	0	2	19
Δrck2	1	0	2	8
Δakl1	0	1	4	13
Δscy1	0	1	1	5
Δmkk1	1	0	1	1
Δkdx1	0	1	0	0
Δkin4	0	0	0	19
Δcmk1	0	0	0	4
Δprr2	0	0	0	2
Δypk2	0	0	0	2
Δpkp2	0	0	0	2
Δkin2	0	0	1	30
Δsmk1	0	0	1	0
Δkkq8	0	0	0	0
Δkcc4	0	0	0	0
Δkin82	0	0	0	0

Phosphatase deletion	Protein up	Protein down	Phosphosite up	Phosphosite down
Δyvh1	847	612	578	496
Δsit4	350	388	860	359
Δppz1	67	163	54	174
Δptc1	99	37	404	44
Δsiw14	20	5	5	2
Δptc7	4	19	12	7
Δoca1	14	9	7	70
Δcmp2	4	18	15	14
Δpph3	4	11	40	7
Δpph22	0	3	45	1
Δppt1	5	2	36	1
Δpph21	1	0	39	0
Δpsr2	6	2	2	1
Δptp3	3	2	2	0
Δpps1	5	0	6	9
Δppz2	4	0	1	0
Δych1	1	3	1	2
Δltp1	3	0	0	4
Δptc5	1	1	3	0
Δptc2	2	0	11	3
Δptp1	1	1	2	2
Δsdp1	0	1	4	0
Δptc3	1	0	13	2
Δcna1	0	1	24	18
Δptc4	0	1	7	10
Δpsr1	0	0	18	5

B

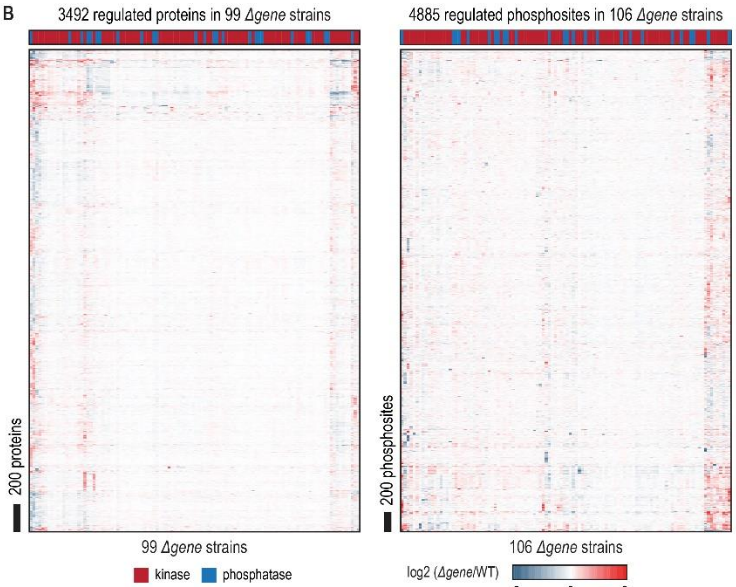

C

	high-impact	medium-impact	low-impact
proteome	12	16	82
phosphoproteome (w/ protein normalization)	9	31	70
overlap	8	10	65

D

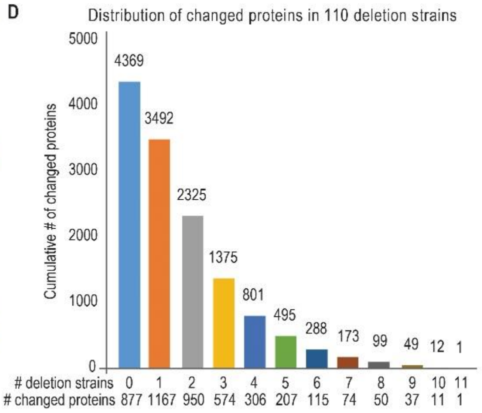

E

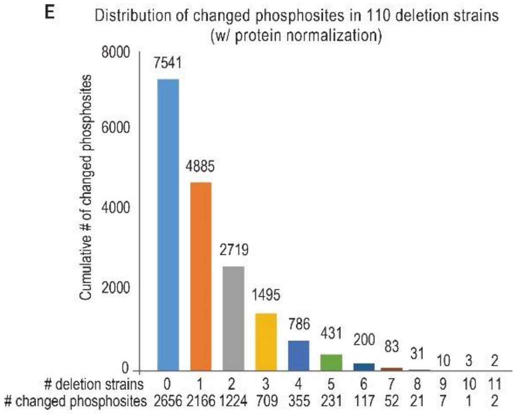

Figure S3. Overview of proteomic and phosphoproteomic phenotypes. Related to Figure 3. (A) Summary of protein changes and phosphosite changes in each kinase (left) and phosphatase (right) deletion strains. **(B)** Hierarchical clustering analysis of deletion strains showing significant perturbed

proteins (left) and phosphosites (right). Missing values were imputed with zero for illustration. **(C)** Numbers of high-impact, medium-impact and low-impact kinases and phosphatases. Kinases or phosphatases disrupting more than 5% of the quantified proteome or phosphoproteome were designated as high-impact, lower than 0.5% are low-impact, between 5% and 0.5% are medium-impact. **(D)** Distribution of disrupted proteins across deletion strains. **(E)** Distribution of perturbed phosphosites across deletion strains.

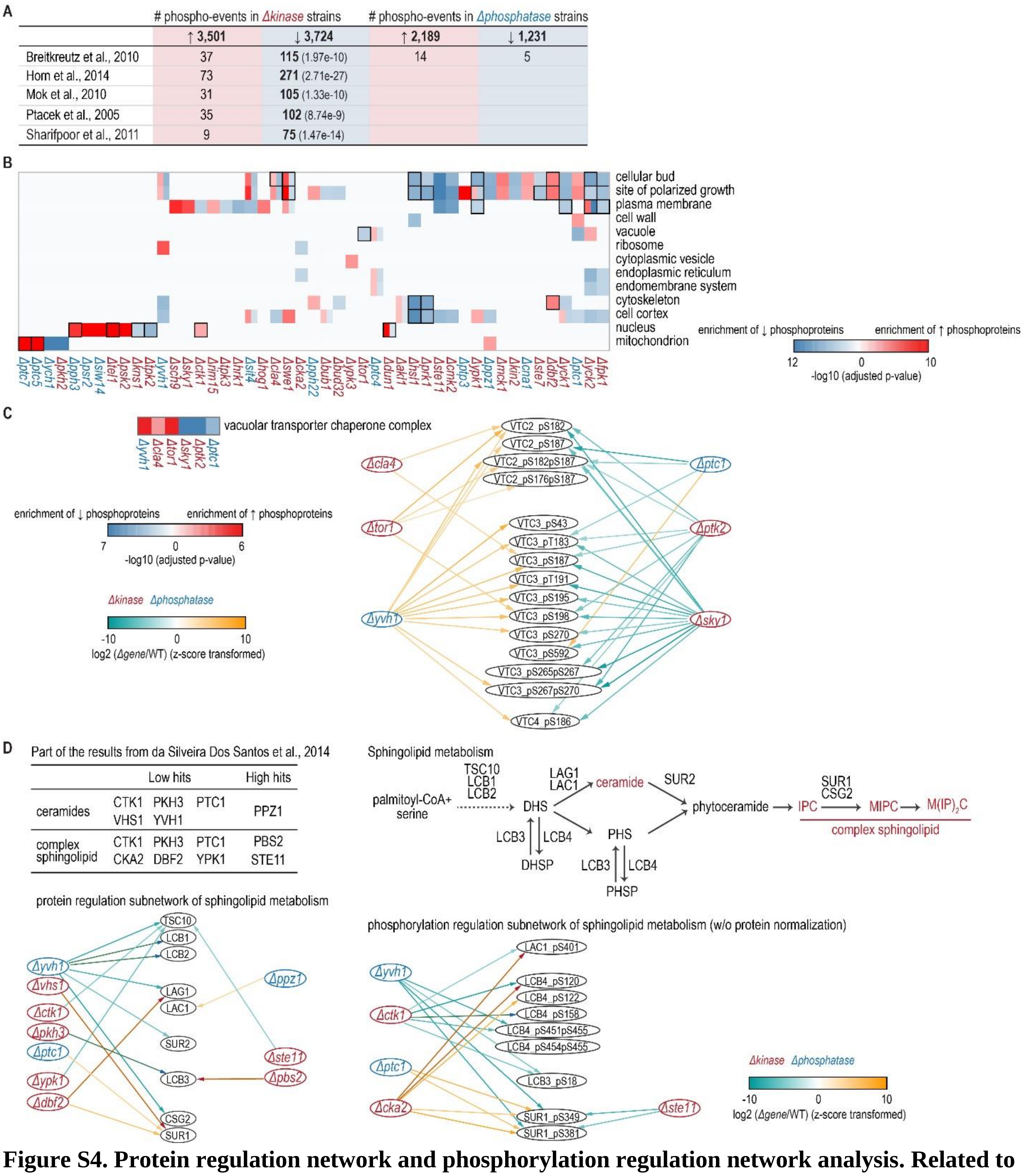

Figure S4. Protein regulation network and phosphorylation regulation network analysis. Related to Figure 4. **(A)** Overlap of changed phosphorylation events between data in this work and other datasets. Numbers inside of parentheses indicate p-values from hypergeometric distribution tests. **(B)** For each kinase and phosphatase, the subcellular localizations enriched in regulated phosphoproteins were computed. Cells with black borders indicate kinases and phosphatases for which known localizations were observed enriched in their phosphoprotein effectors. **(C)** Example of functionally related kinases and phosphatases in regulating phosphorylation status of vacuolar transporter chaperone complex. **(D)** Regulation of sphingolipid metabolism. All changed sphingolipid metabolism enzymes and their

regulators were extracted from protein regulation network and phosphorylation regulation network (without protein normalization) to explain phenotypes observed in da Silveira Dos Santos et al., 2014.

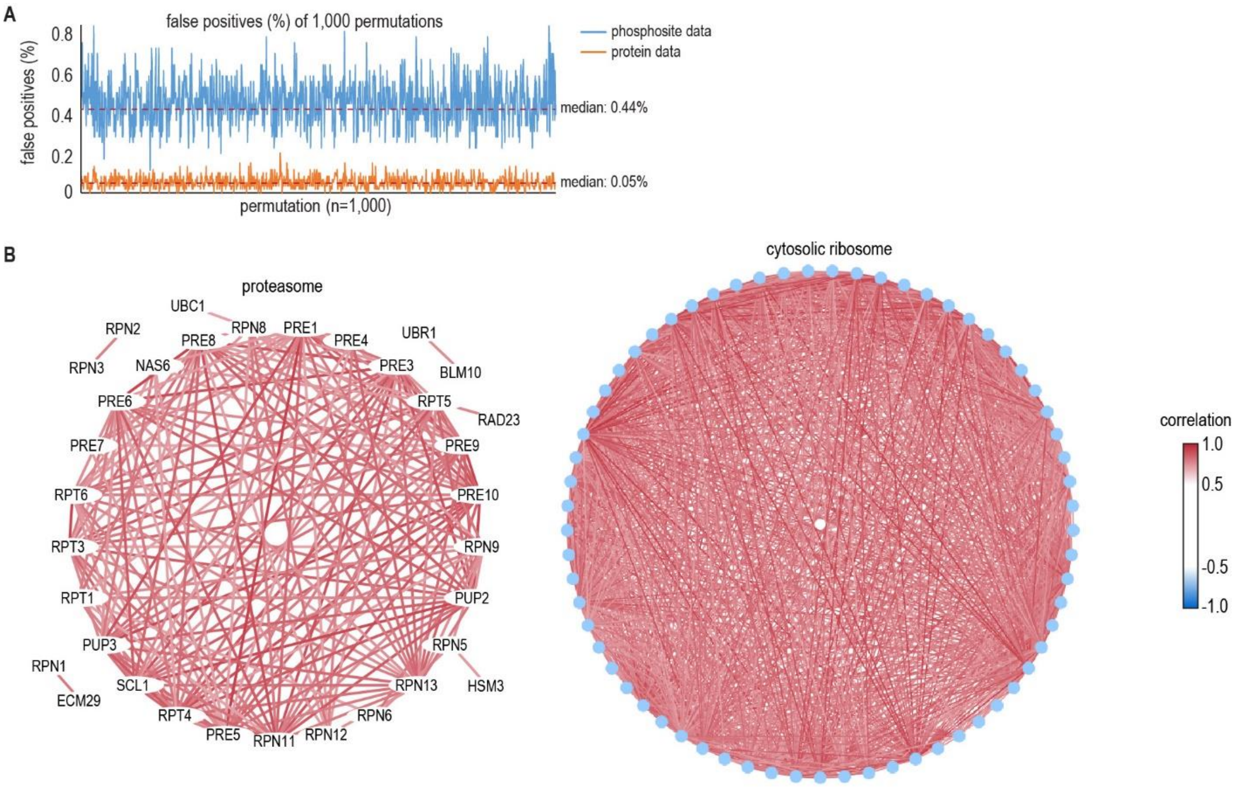

Figure S5. Protein covariance network analysis. Related to Figure 6. (A) With |r| ≥ 0.7 and Bonferroni-adjusted p ≤ 0.001, permutation tests showed 0.05% and 0.44% false positives for protein covariance network and phosphosite covariance network, respectively. **(B)** Protein covariance network analysis recapitulated proteasome and cytosolic ribosome.

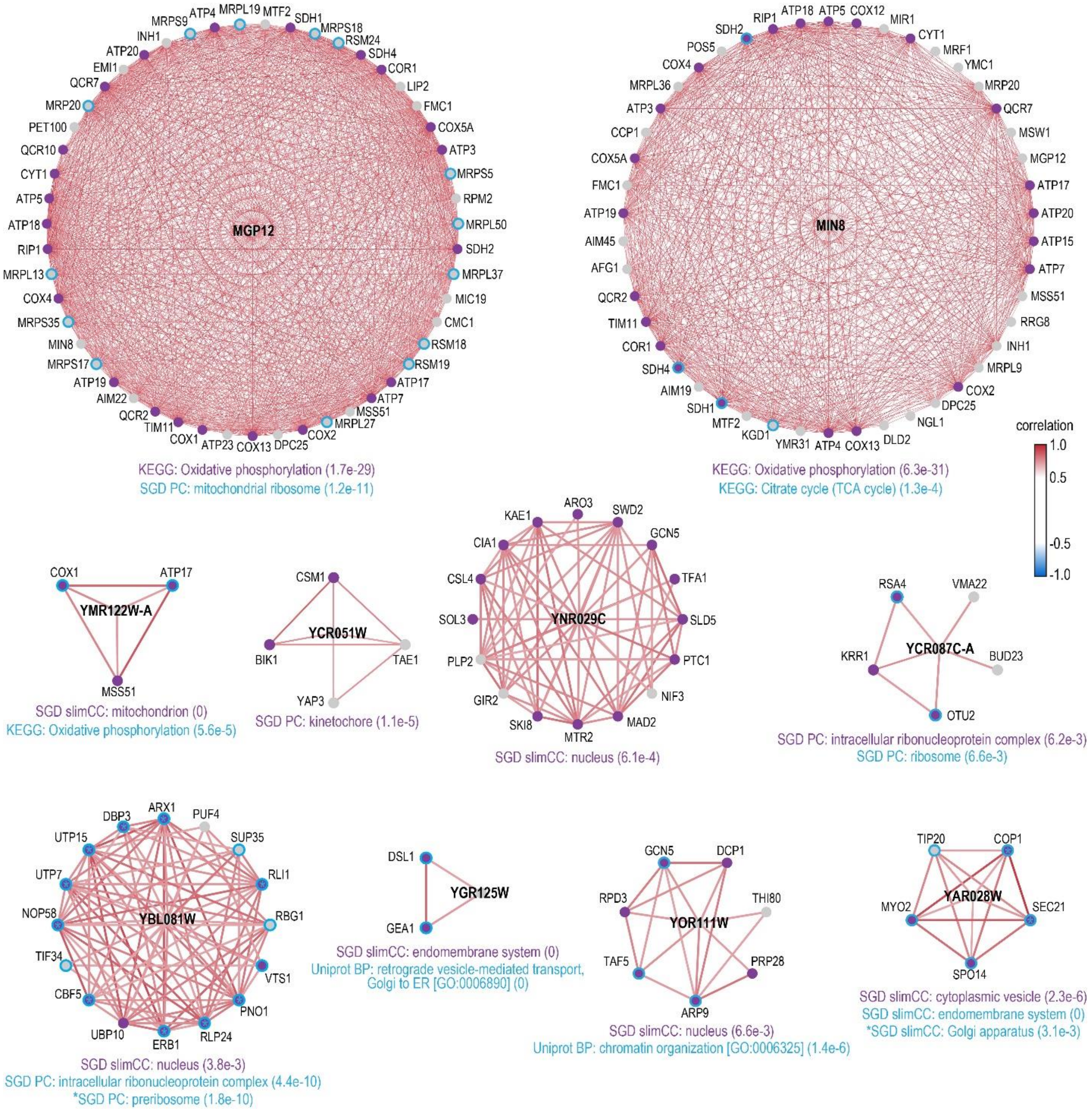

Figure S6. Examples of predications that can be generated from neighbor protein covariance networks. Related to Figure 6. For a given protein, neighbors were tested for GO term enrichment with Benjamini-Hochberg adjustment to account for multiple hypothesis testing.

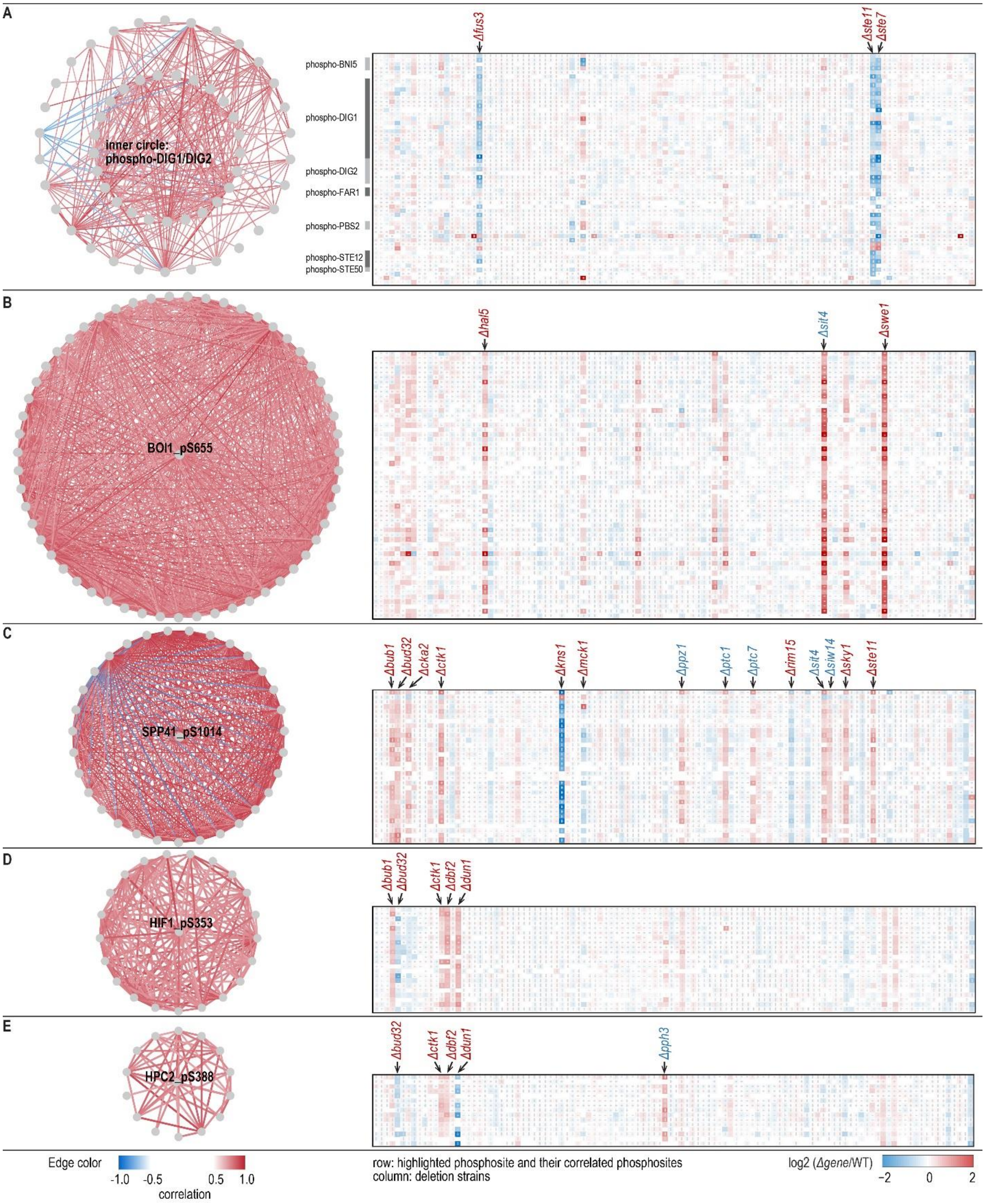

Figure S7. Phosphosite covariance network analysis. **Related to Figure 7. (A)** Phosphorylated DIG1 and DIG2 centered subnetwork recapitulated known target proteins (BNI5, FAR1, PBS2, STE12 and STE50) and architecture of pheromone responsive pathway (STE7, STE11 and FUS3). (**B-E**) For a given phosphosite, its covariant phosphosites and potential kinases and phosphatases coordinately modulating

their dynamics could be inferred from phosphosite covariance network. Examples of 4 phosphosites are shown here.
